# Stage-specific functions of Semaphorin7A during adult hippocampal neurogenesis rely on distinct receptors

**DOI:** 10.1038/ncomms14666

**Published:** 2017-03-10

**Authors:** Bart C. Jongbloets, Suzanne Lemstra, Roberta Schellino, Mark H. Broekhoven, Jyoti Parkash, Anita J. C. G. M. Hellemons, Tianyi Mao, Paolo Giacobini, Henriette van Praag, Silvia De Marchis, Geert M. J. Ramakers, R. Jeroen Pasterkamp

**Affiliations:** 1 Department of Translational Neuroscience, Brain Center Rudolf Magnus, University Medical Center Utrecht, 3584 CG Utrecht, The Netherlands; 2 Dipartimento di Scienze della Vita e Biologia dei Sistemi and Neuroscience Institute Cavalieri Ottolenghi, University of Torino, 10100 Torino, Italy; 3 Centre for Animal Sciences, School of Basic and Applied Sciences, Central University Punjab, City Campus, Mansa Road, Bathinda 151001, India; 4 Vollum Institute, Oregon Health & Science University, Portland, Oregon 97239, USA; 5 Inserm, Laboratory of Development and Plasticity of the Neuroendocrine Brain, Jean-Pierre Aubert Research Centre, U1172, 59045 Lille, France; 6 University of Lille, 59045 Lille, France; 7 Neuroplasticity and Behavior Unit, Laboratory of Neurosciences, Intramural Research Program, National Institute on Aging, National Institutes of Health, Baltimore, Maryland 21224, USA

## Abstract

The guidance protein Semaphorin7A (Sema7A) is required for the proper development of the immune and nervous systems. Despite strong expression in the mature brain, the role of Sema7A in the adult remains poorly defined. Here we show that Sema7A utilizes different cell surface receptors to control the proliferation and differentiation of neural progenitors in the adult hippocampal dentate gyrus (DG), one of the select regions of the mature brain where neurogenesis occurs. PlexinC1 is selectively expressed in early neural progenitors in the adult mouse DG and mediates the inhibitory effects of Sema7A on progenitor proliferation. Subsequently, during differentiation of adult-born DG granule cells, Sema7A promotes dendrite growth, complexity and spine development through β1-subunit-containing integrin receptors. Our data identify Sema7A as a key regulator of adult hippocampal neurogenesis, providing an example of how differential receptor usage spatiotemporally controls and diversifies the effects of guidance cues in the adult brain.

## Abstract (web summary)

The functions of semaphorins in the adult brain are poorly understood. Here the authors show that Sema7A carries out stage-specific functions in the adult hippocampus via differential receptor usage; in progenitor cells, Sema7A inhibits proliferation via acting on PlexinC1, whereas in adult-born neurons, it promotes dendrite growth through β1-integrins.

Semaphorins comprise a large family of extracellular proteins that provide guidance to growing neurites and migrating cells during development^1^,^2^. Many semaphorins are prominently expressed in the embryonic nervous system, but their expression generally declines and becomes more restricted at adult stages. Semaphorin7A (Sema7A), the only glycophosphatidylinositol-linked member of the semaphorin family, is one of few semaphorins or guidance cues in general, that display markedly increased expression as the central nervous system (CNS) matures^3^,^4^. However, while the developmental roles of Sema7A have been well-characterized^5^,^6^,^7^,^8^,^9^,^10^, the precise function of Sema7A in the adult nervous system remains poorly understood^11^.

Two cell surface receptors mediate the biological effects of Sema7A: heterodimeric integrin receptors, composed of β1 and α1 or αV subunits, and plexinC1 (also known as VESPR or CD232) (ref. 12). β1-subunit-containing integrins serve as well-characterized neuronal receptors for Sema7A (ref. 5), but also transduce the non-neuronal effects of Sema7A for example in the immune system^13^,^14^. Much less, however, is known about the role of plexinC1. Sema7A binds plexinC1 (refs 15, 16) and in neurons Sema7A-plexinC1 signalling regulates synapse development and neuroglia plasticity^6^,^11^. Studies focusing on immune system function or cancer indicate roles for plexinC1 in cell migration and proliferation^17^,^18^,^19^, but whether plexinC1 exerts similar cell biological effects in the CNS, and if so, whether these require Sema7A, remains to be determined.

In the adult CNS, cell proliferation is limited to two neurogenic regions, the subventricular zone (SVZ) of the lateral ventricles and the subgranular zone (SGZ) of the hippocampal dentate gyrus (DG). These neurogenic regions harbour adult neural progenitor cells (aNPCs), which give rise to astrocytes, intermediate progenitor cells (IPCs, also known as amplifying neural progenitors), and both immature and mature granule cells (GCs). Cell proliferation in the adult SVZ and SGZ is tightly controlled to avoid exhaustion of the neurogenic aNPC pool. This involves regulation of the balance between quiescent and active aNPCs and strict gating of the number of actively proliferating cells^20^,^21^,^22^. Interestingly, recent work reveals expression of plexinC1 in aNPCs within the SVZ^23^, suggesting that plexinC1 may contribute to aNPC proliferation regulation.

New-born GCs in the adult brain (hereafter referred to as adult-born granule cells, or nGCs) need to integrate in a fully developed neural circuit. Although many of the key factors required for the regulation of aNPC proliferation in the SGZ have been identified, much less is known about the extracellular molecular signals that control the morphological development and integration of nGCs. Here we report that, in the adult mouse hippocampus, expression of plexinC1 is restricted to the SGZ and is largely confined to early progenitor cells. In line with this pattern of expression, Sema7A suppresses progenitor proliferation through plexinC1 *in vitro*, and *Sema7A* and *plexinC1* knockout mice display identical cell proliferation defects in the SGZ *in vivo*. At subsequent stages of adult neurogenesis, Sema7A is required for nGC dendrite outgrowth, spine development and functional integration of excitatory inputs to the nGCs. Remarkably, our data suggest that these morphological effects require β1-integrins, but not plexinC1. Together, our data reveal a novel role for Sema7A in the adult brain, implicate semaphorins and plexins in the control of cell proliferation during adult hippocampal neurogenesis, and provide an example of how a single guidance cue can regulate multiple stages of adult neurogenesis via two, stage-specific, distinct receptors.

## Results

### PlexinC1 is confined to the neurogenic niche of the adult DG

**Figure 1:**
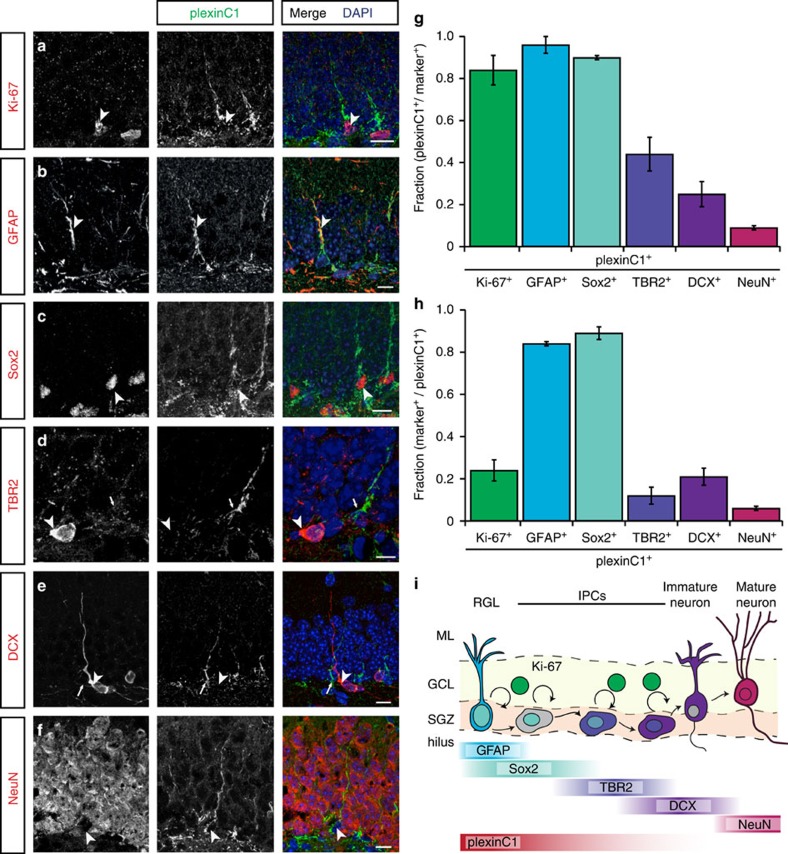
PlexinC1 expression in the subgranular zone is largely confined to early progenitors. (**a**–**f**) Double immunohistochemistry for plexinC1 and Ki-67 (**a**), glial fibrillary acidic protein (GFAP; **b**), sex determining region Y-box 2 (Sox2; **c**), t-box brain 2 (TBR2; **d**), doublecortin (DCX; **e**) or neuronal nuclei (NeuN; **f**) in sections of the adult mouse dentate gyrus (DG). The majority of plexinC1-positive cells express Ki-67, GFAP and Sox2 but not TBR2, DCX or NeuN. Arrowheads indicate cells expressing specific markers. Arrows indicate plexinC1-positive cells not expressing the indicated marker. (**g**,**h**) Quantification of the fraction of cells expressing a specific marker that also express plexinC1 (**g**) or the fraction of plexinC1-positive cells that also express the indicated marker protein (**h**). Data are presented as means±s.e.m. *n*≥3 (mice). (**i**) Schematic representation of lineage-marker and plexinC1 expression in cell lineage subtypes during neuronal differentiation in the adult DG. Ki-67 is a marker for proliferating cells during all active phases of the cell cycle. PlexinC1 is expressed in GFAP-positive and Sox2-positive radial glia-like cells (RGLs) and early intermediate progenitor cells (IPCs). Ki-67-positive proliferating cells express plexinC1. In contrast, only a small fraction of TBR2-, DCX- or NeuN-positive intermediate progenitor cells and (immature) granule cells express plexinC1. Scale bars: 10 μm.

Expression of *Sema7A* in the brain increases as development progresses^3^. However, the precise role of this and the expression of many other semaphorins in the adult brain remains incompletely understood. Sema7A exerts its biological effects via two unrelated receptors, plexinC1 and β1-subunit-containing integrins^12^. Whereas expression of β1-integrins in the mature rodent brain is rather strong and ubiquitous^24^, expression of *plexinC1* is much more restricted^3^. Therefore, to unravel specific function(s) of Sema7A in the adult brain we first focused on plexinC1 and performed immunohistochemistry for this receptor at adult stages. In line with our previous work showing restricted *plexinC1* expression in the adult mouse brain^3^, plexinC1 immunolabelling was detected in a small number of brain regions, including the olfactory bulb, rostral migratory stream (RMS), cerebellum and DG of the hippocampus (Supplementary Fig. 1a–g;). Mature GCs in the DG are lined up within the granular cell layer (GCL) and project their axons to the CA3 region of the hippocampus. Beneath the GCL is a layer termed the SGZ, which harbours proliferating neuronal progenitor cells (aNPCs). aNPCs give rise to nGCs throughout adult life (Fig. 1i). Immunohistochemistry showed that expression of plexinC1 in the DG GCL is confined to cells located in the SGZ (Supplementary Fig. 1c). In contrast to the specific expression of plexinC1, Sema7A was expressed throughout the adult DG (Supplementary Fig. 1h–j). The SGZ consists of aNPCs, such as radial glia-like cells (RGLs) and multiple types of IPCs, as well as immature nGCs (Fig. 1i). To reveal which cells in the SGZ express plexinC1, we performed double and triple immunohistochemistry experiments with several cell-type specific markers including Ki-67, GFAP and Sox2 (Fig. 1, Supplementary Fig. 2). Ki-67 labels proliferating cells and most Ki-67^+^ cells showed plexinC1 expression (Fig. 1a,g,h). However, plexinC1 expression was not restricted to proliferating cells, because only a small fraction of all plexinC1^+^ cells expressed Ki-67 (Fig. 1h). RGLs, which express GFAP and Sox2 (Fig. 1b,c), are recognizable by their short process extending into the GCL (ref. 25). This morphology is reminiscent of the majority of plexinC1^+^ cells in the SGZ (Fig. 1a–f, Supplementary Figs 1c and 2a–e). Indeed, co-labelling showed that most GFAP^+^ and Sox2^+^ cells express plexinC1, and also that most plexinC1-expressing cells displayed GFAP and Sox2 immunolabelling (Fig. 1b,c,g,h, Supplementary Figs 1c and 2a,c,d,f). About half of these cells were proliferating as evidenced by Ki-67 expression (Supplementary Fig. 2c,d,g). Only a small fraction of T-box brain protein (TBR2)-positive IPCs (Fig. 1d,g, Supplementary Fig. 2b,f) or doublecortin (DCX)-positive IPCs and immature nGCs (Fig. 1e,g, Supplementary Fig. 2b,f) expressed plexinC1, and we found that only a small subset of plexinC1^+^ cells displayed TBR2 or DCX labelling (Fig. 1h, Supplementary Fig. 2f,g). Finally, most NeuN^+^ mature GCs did not express plexinC1, while plexinC1^+^ cells only rarely displayed NeuN signals (Fig. 1f–h). Together, these results indicate that in the neurogenic niche of the DG plexinC1 is mostly confined to early progenitor cells, including RGLs and early IPCs, independent of their proliferation state (Fig. 1i).

### Sema7A inhibits proliferation of aNPCs through plexinC1

**Figure 2:**
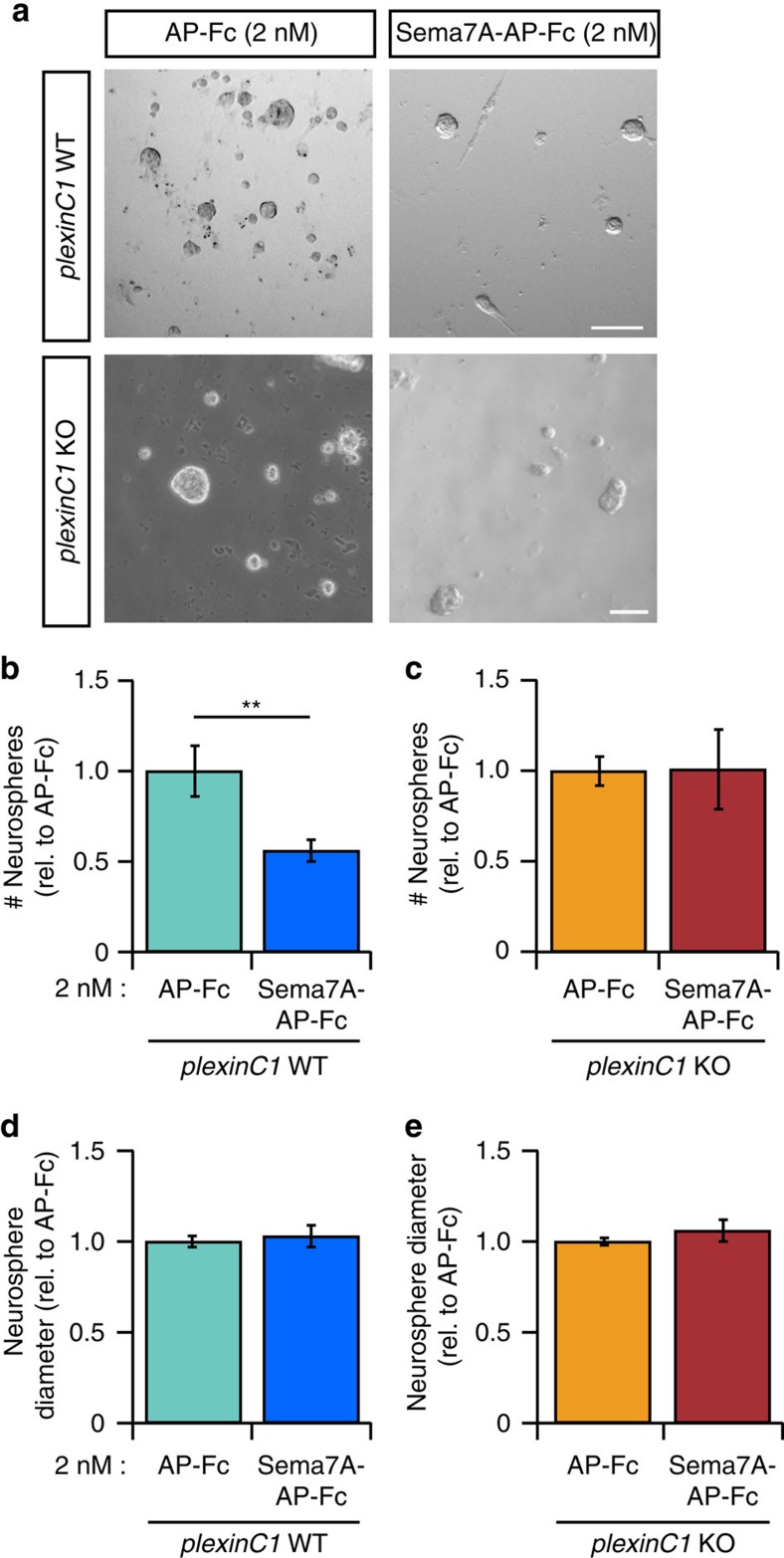
Sema7A inhibits neurosphere formation from adult SGZ-isolated neuronal progenitors through plexinC1. (**a**) Application of exogenous Sema7A-AP-Fc to *plexinC1*^*+/+*^(WT) aNPCs for 8 days *in vitro* reduces neurosphere number compared to AP-Fc control treatment. This reduction is lost in *plexinC1*^*−/−*^ (KO) aNPCs. (**b**–**e**) Quantification of WT neurosphere growth in the presence of Sema7A-AP-Fc reveals a significant reduction in neurosphere number (replicate-paired Student's *t*-test; **b**) but not diameter (*P*=0.0674, *n*=5 mice; **d**). aNPCs from KO mice are not sensitive to the proliferation inhibiting effects of Sema7A-AP-Fc (replicate-paired Student's *t*-test: *P*_(diameter)_=0.9484, *P*_(number)_=0.7738, *n*=5 mice; **c**,**e**). Data are presented as means±s.e.m., **P<0.01. Scale bar: 100 μm.

The specific expression of plexinC1 in aNPCs (Fig. 1) led us to hypothesize that Sema7A and plexinC1 regulate cell proliferation during adult neurogenesis. To examine this model, we performed experiments with neurosphere cultures. Proliferation of aNPCs in dissociated *in vitro* suspension culture leads to the formation of neurospheres, which express *plexinC1* (Supplementary Fig. 3a). Neurosphere size and number are routinely used as a measure for aNPC proliferation^26^. We first assessed whether Sema7A could modulate aNPC proliferation. Indeed, application of 2 nM Sema7A-AP-Fc to wild-type aNPCs for 8 days strongly reduced the number of neurospheres (Fig. 2a,b,d; *n*=5 mice, Student's *t*-test: *P*=0.0172), as compared to control (AP-Fc, 2 nM). In contrast, the number and diameter of neurospheres formed from *Sema7A*^*−/−*^ aNPCs resembled *Sema7A*^*+/+*^ control (Supplementary Fig. 3b,c; *n*=5 mice, Student's *t*-test: *P*_(number)_*=*0.4515, *P*_(diameter)_=0.8900). To determine whether exogenous Sema7A affects aNPC proliferation via plexinC1, *plexinC1*^*−/−*^ aNPCs were exposed to Sema7A. Application of Sema7A reduced the number of neurospheres derived from *plexinC1*^*+/+*^ but not *plexinC1*^*−/−*^ aNPCs (Fig. 2a–e; *n*=5 mice, Student's *t*-test: *P*_(number)_=0.7738, *P*_(diameter)_=0.9484). Genetic depletion of plexinC1 by itself did not affect neurosphere number (*n*=3 mice, *plexinC1*^*+/+*^=1.000±0.0156, *plexinC1*^*−/−*^=1.005±0.1383, Student's *t*-test: *P*=0.9712 (mean normalized to *plexinC1*^*+/+*^±s.e.m.)). Together, these results suggest that Sema7A-plexinC1 signalling negatively regulates aNPC proliferation *in vitro*.

### Sema7A-plexinC1 inhibit proliferation in the adult DG *in vivo*

**Figure 3:**
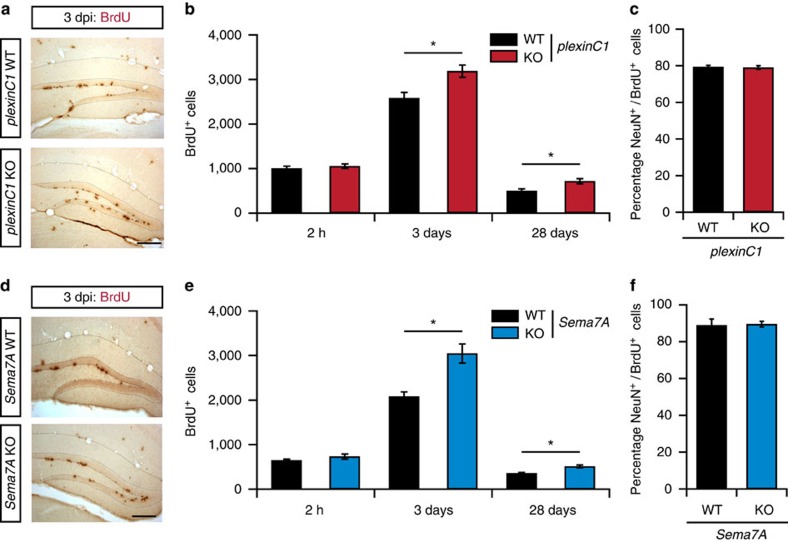
*PlexinC1*^*−/−*^ and *Sema7A*^*−/−*^ mice display increased cell proliferation in the adult DG. (**a**,**d**) Representative examples of BrdU immunostaining in the dentate gyrus (DG) of adult wild-type littermates (WT), *plexinC1*^*−/−*^ or *Sema7A*^*−/−*^ mice at 3 days after BrdU injection (dpi). KO, knockout. (**b**,**e**) Adult mice were injected with BrdU and processed for immunohistochemistry for BrdU at 2 h, 3 days and 28 days post-injection. An increase in the number of BrdU-positive cells was detected at 3 and 28 dpi in *plexinC1*^*−/−*^ and *Sema7A*^*−/−*^ mice as compared to WT (two-way ANOVA, *post-hoc t*-test, 2 h: *n*_(WT, *plexinC1*_^*−/−*^)=4, *n*_(*Sema7A*_^*−/−*^)=3, 3 days: *n*_(WT, *Sema7A*_^*−/−*^)*=4, n*_(*plexinC1*_^*−/−*^)=7, 28 days: *n*_(*plexinC1*_^*−/−*^,_-WT)_=5, *n*_(*Sema7A*_^*−/−*^)=4, *n*_(*Sema7A*-WT)_=3). (**c**,**f**) Quantification of the number NeuN/BrdU-positive cells in the indicated mouse models at 28 dpi (Wilcoxon-Mann-Whitney test, Sema7A: *n*_(Ki-67, DCX)_=4, *plexinC1*: *n*_(WT: Ki-67, DCX)_*=3, n*_(*plexinC1*_^*−/−*^_: Ki-67, DCX)_*=4*, *P*_(*plexinC1*)_=0.8728, *P*_(*Sema7A*)_=0.7114). Data are presented as means±s.e.m., **P<0.01, *P<0.05. Scale bars: 250 μm.

To confirm these *in vitro* observations and to examine whether Sema7A and plexinC1 can regulate aNPC proliferation *in vivo*, bromodeoxyuridine (BrdU)-pulse labelling was performed in adult mice. Proliferating cells incorporate BrdU during DNA-replication allowing quantification of the number of proliferating cells^27^. At 2 h after a BrdU injection, the number of BrdU^+^ cells in the DG was similar in *plexinC1*^*+/+*^ and *plexinC1*^*−/−*^ mice (Fig. 3a,b; *n*=4 mice, *post-hoc t*-test: *P*_(2 h)_=0.335; Supplementary Fig. 5a–d). In contrast, the number of BrdU^+^ cells in *plexinC1*^*−/−*^ mice was significantly increased 3 and 28 days after BrdU injections (Fig. 3a,b; *n*≥4 mice, two-way ANOVA: genotype × time F_(2,15)_=0.003, *post-hoc t*-test: *P*_(3 days)_=0.0208, *P*_(28 days)_=0.0208). To confirm these data, immunohistochemistry for Ki-67 and DCX was performed. In line with the BrdU data, quantification showed an increase in Ki-67^+^ proliferating cells and DCX^+^ immature nGCs in adult *plexinC1*^*−/−*^ mice (Supplementary Fig. 6a,b; *n*≥3 mice, Student's *t*-test: *P*_(Ki-67)_=0.0445, *P*_(DCX)_=0.0121). These data, together with the selective expression of plexinC1 in the SGZ (Fig. 1), our *in vitro* observations (Fig. 2) and previous work revealing an inhibitory effect of plexinC1 on cell proliferation^18^, suggest that plexinC1 negatively regulates aNPC proliferation *in vivo*. In addition, quantification revealed that the relative number of differentiated (NeuN^+^) BrdU^+^ cells was similar in *plexinC1*^*+/+*^ and *plexinC1*^*−/−*^ mice (Fig. 3c; *n*=3 mice, Student's *t*-test: *P*=0.8728). This suggests that plexinC1 is not required for the differentiation of aNPCs into nGCs.

Because Sema7A strongly reduced neurosphere formation via plexinC1 and *plexinC1*^*−/−*^ mice showed enhanced proliferation, we next examined aNPC proliferation in adult *Sema7A*^*−/−*^ mice. Analogous to our observations in *plexinC1*^*−/−*^ mice, no changes in the number of BrdU^+^ cells were found at 2 h after injection in *Sema7A*^*−/−*^ mice (Fig. 3d,e; *n*≥3 mice, *post-hoc t*-test: *P*=0.5141), while significantly more BrdU^+^ cells were detected at 3 and 28 days post-injection (dpi, Fig. 3d,e; *n*≥3 mice, two-way ANOVA: genotype × time F_(2,22)_=0.0129, *post-hoc t*-test: *P*_(3 days)_=0.0313, *P*_(28 days)_=0.0313). Quantification of Ki-67^+^ and DCX^+^ cells confirmed these results, showing an increase in proliferating cells and immature nGCs in the adult DG of *Sema7A*^*−/−*^ mice (Supplementary Fig. 6c,d; *n*=4 mice, Student's *t*-test: *P*_(Ki-67)_=0.0279, *P*_(DCX)_=0.0294). In contrast, no change in the number of BrdU^+^ cells expressing NeuN was observed (Fig. 3f; *n*=3 mice, Student's *t*-test: *P*=0.7114). Together, these results indicate that Sema7A inhibits aNPC proliferation in the adult DG.

Next we addressed the consequence of the increased aNPC proliferation observed in *plexinC1*^*−/−*^ and *Sema7A*^*−/−*^ mice. Increased proliferation could lead to an increase in GC density in the DG, faster depletion of the aNPC pool and/or a larger pool of immature cells during aging^28^. To assess the specific effect of loss of Sema7A-plexinC1 signalling, we focused on *plexinC1*^*−/−*^ mice. First, we observed that GC density in the DG was unchanged but found an increase in the number of cleaved caspase-3^+^ cells in the mature DG of *plexinC1*^*−/−*^ mice (Supplementary Fig. 6e–g; *n*_(cell density)_=3 and *n*_(Caspase3)_=6 mice, Student's *t*-test: *P*_(cell density)_=0.141, *P*_(Caspase3)_=0.039). Second, we analysed aNPC proliferation at older ages to examine aNPC pool depletion. BrdU injection and quantification at 3 dpi in ageing *plexinC1*^*−/−*^ mice (2, 6 and 18 months of age) revealed a comparable decrease in the number of BrdU^+^ cells during ageing. However, at every age examined a larger number of BrdU^+^ cells were detected in *plexinC1*^*−/−*^ mice as compared to WT littermates (Supplementary Fig. 6h; *n*≥3 mice, two-way ANOVA: genotype × time F_(2,19)_=0.0283, *post-hoc t*-test: *P*_(2 months)_=0.0469, *P*_(6 months)_=0. 0469, *P*_(18 months)_=0. 0469). Third, the number of GFAP^+^ RGL cells and DCX^+^ cells, that is, late progenitor cells and immature GCs, was also elevated at 18 months of age in *plexinC1*^*−/−*^ mice (Supplementary Fig. 6i,j; *n*=3 mice, Student's *t*-test: *P*_(GFAP)_=0.036 *P*_(DCX)_=0.023). Thus, loss of plexinC1 leads to an increased number of progenitor cells and immature GCs at older age and triggers caspase-3-dependent apoptosis without altering overall cell density in the adult DG.

Several of the reported Sema7A effects on neurons are mediated by β1-subunit-containing integrin receptors. β1-integrins are expressed in the SGZ of the DG and have been implicated in stem cell proliferation and survival (Supplementary Fig. 7a–c)^24^,^29^,^30^,^31^. To determine whether β1-integrins cooperate with plexinC1 to mediate the effects of Sema7A on aNPCs, BrdU-pulse labelling was performed in *β1-integrin* knockout mice. Null mutant *β1-integrin* mice do not survive into adulthood, so we generated neural-specific *β1-integrin* knockout mice by crossing *nestin-Cre* with *β1-integrin*^*fl/fl*^ mice. As reported previously, adult *β1-integrin*^*fl/fl*^;*nestin-Cre* mice displayed cortical layering defects, subtle changes in the organization of the DG infrapyramidal blade and abnormal positioning and orientation of a small subset of DCX^+^ cells (Supplementary Fig. 7d–f)^32^,^33^. However, we did not detect significant changes in the number of BrdU^+^ cells in *β1-integrin*^*fl/fl*^;*nestin-Cre* mice at 2 h and 3 days after BrdU injection (Supplementary Fig. 7g,h; *n*=3, Student's *t*-test: *P*_(2 h)_=0.8302 *P*_(3 days)_=0.4199). Similarly, the number of Ki-67^+^ cells in the SGZ of *β1-integrin*^*fl/fl*^;*nestin-Cre* mice was normal (Supplementary Fig. 7i; *n*≥8 mice, Student's *t*-test: *P*=0.1064). These results contrast with our observations in *plexinC1*^*−/−*^ and *Sema7A*^*−/−*^ mice, which displayed changes in BrdU^+^ and Ki-67^+^ cell number at 3 dpi. While proliferation appeared intact in *β1-integrin*^*fl/fl*^;*nestin-Cre* mice, neural-specific ablation of *β1-integrin* caused a pronounced increase in DCX^+^ cell number in the DG (Supplementary Fig. 7j; *n*=3 mice, Student's *t*-test: *P*=0.0270). Thus, although β1-integrins do indeed affect specific aspects of adult neurogenesis, the early role of Sema7A during aNPC proliferation that we observe appears to be independent of β1-integrins.

### Dendritic development of nGCs requires Sema7A

**Figure 4:**
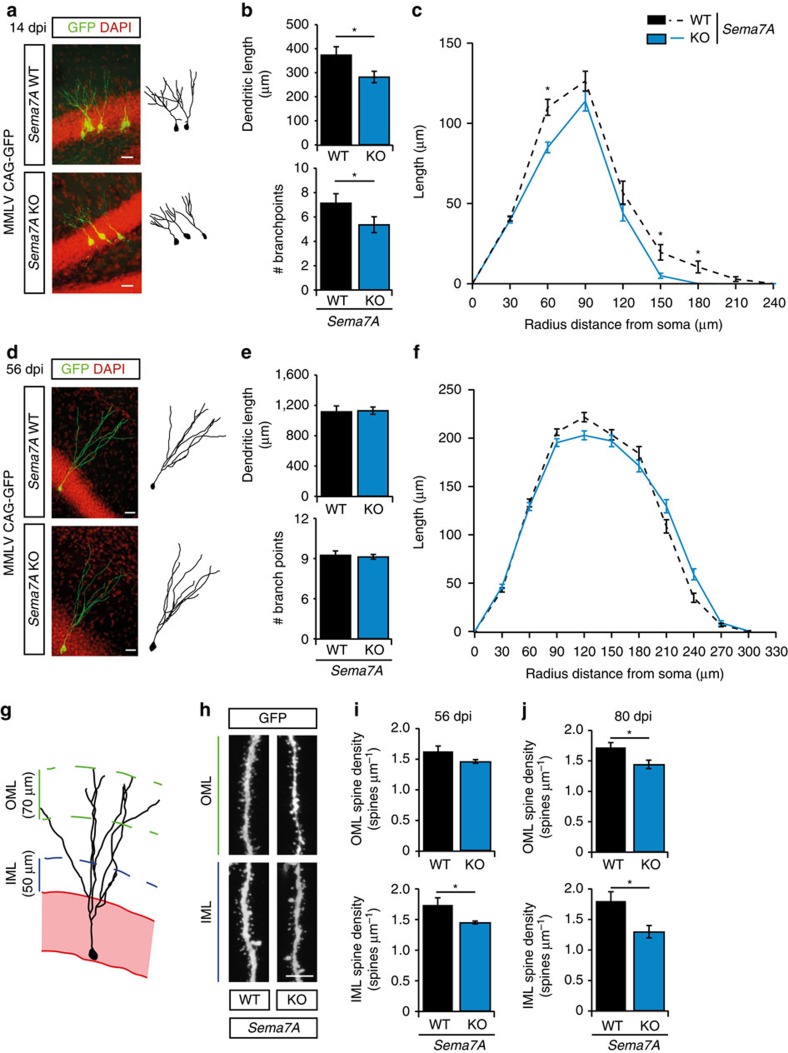
Sema7A is required for dendritic growth and spine development in adult-born DG granule cells. (**a**,**d**) The dentate gyrus of adult *Sema7A*^*+/+*^ (WT) or *Sema7A*^*−/−*^ (KO) mice was injected with MMLV CAG-GFP to label adult-born granule cells and immunostained for GFP at 14 or 56 days post-injection (dpi). DAPI is shown in red. Right panels depict representative tracings based on confocal z-stack images. (**b**,**e**) Quantification of dendritic length and number of branch points in WT (black bars) and KO (blue bars) littermates (two-way ANOVA, *post-hoc t*-test, 14 dpi: *n*=3, 56 dpi: *n*=4, *P*_(56 dpi: length)_=0.150 and *P*_(56 dpi: branch points)_=0.868). (**c**,**f**) Sholl analysis of the dendritic tree of GFP-labelled cells in WT (black dashed-lines) and KO mice (blue lines). Dendritic length per radius distance from soma (30 μm radius interval) at 14 dpi, but not at 56 dpi, was decreased in KO mice (14 dpi: *n*=117 WT cells/3 mice, *n*=120 KO cells/3 mice, 56 dpi: *n*=134 WT cells/4 mice, *n*=151 KO cells/4 mice. Repeated-measures ANOVA 14 dpi: F_(7, 1645)_=3.219, 56 dpi: F_(9, 1224)_=2.2560, *P*=0.056). (**g**) Schematic representation of the DG indicating the regions selected for spine density quantification. IML, inner molecular layer; OML, outer molecular layer. (**h**) Representative images showing spine-bearing dendritic segments in the OML and IML in WT and KO mice. (**i**) Quantification of spine density on adult-born granule cells (nGCs) at 56 dpi shows differences between WT (*n*=5) and KO (*n*=5) mice in the IML (*post-hoc t*-test: *P*_(OML)_=0.081). (**j**) Quantification of spine density on nGCs at 80 dpi shows differences between WT (*n*=3) and KO (*n*=3) mice in the IML and OML (Student's *t*-test). Data are presented as means±s.e.m. *P<0.05. Scale bars: (**a**,**d**) 25 μm; (**h**) 10 μm.

Expression of Sema7A in the adult DG is not restricted to the SGZ but is also found in more superficial layers of the DG that contain immature nGCs and their dendrites. This observation, together with the fact that Sema7A can regulate dendritic growth of embryonic neurons^10^, prompted us to examine whether Sema7A controls dendrite development in nGCs. Retroviral labelling of proliferating cells (aNPCs) with Moloney murine leukaemia virus (MMLV)-based retroviral vectors allows for tracing and analysis of the dendritic morphology of nGCs (refs 34, 35, 36). Adult *Sema7A*^*−/−*^ mice and littermate controls were injected with MMLV CAG-GFP in the DG to selectively visualize nGCs (Fig. 4a). Quantification of the position of GFP^+^ nGC cell bodies relative to the SGZ, a measure of nGC migration, at 14 dpi did not reveal significant differences between wild-type and mutant littermates (Supplementary Fig. 8a–c; littermate-paired Student's *t*-test *n*=4 mice, *P*_(distance)_=0.1645, *P*_(relative distance)_=0.2246). However, analysis of dendritic morphology revealed a reduction in total dendritic length and the number of dendritic branch points in GFP^+^ nGCs in *Sema7A*^*−/−*^ mice (Fig. 4a,b; *n*=3 mice, *post-hoc t*-test: *P*_(dendritic length)_=0.045 and *P*_(branch points)_=0.0499). Sholl-analysis confirmed this reduction in dendritic length and branch points (Fig. 4c, Supplementary Fig. 8f; *n*=3 mice, repeated-measures ANOVA: Sholl length; F_(7, 1,645)_=2.800, *P*=0.045, Sholl intersections; F_(7, 1,645)_=3.219, *P*=0.025). This effect of Sema7A ablation was no longer observed at 56 dpi (Fig. 4d–f, Supplementary Fig. 8g; *n*=4 mice, two-way ANOVA, dendritic length: dpi F_(1, 10)_=205.078, *P*<0.001, *post-hoc t*-test: *P*_(dendritic length)_=0.150, branchpoints: dpi F_(1, 10)_=12.448, *P*=0.006, *post-hoc t*-test: *P*_(branch points)_=0.868, repeated-measures ANOVA: Sholl length; F_(9, 1,224)_=2.560, *P*=0.056, Sholl intersections; F_(9, 1,224)_=2.202, *P*=0.082). These data indicate that Sema7A is required for the initial phase of dendritic outgrowth and ramification of nGCs in the adult DG.

### Sema7A controls dendritic spine number in nGCs

Although the role of Sema7A in the adult brain remains poorly characterized, recent work implicates this semaphorin in the regulation of dendritic spines in the postnatal cerebellum^6^. We therefore explored the effect of Sema7A ablation on dendritic spine density in nGCs in the adult DG. Dendritic spines begin to emerge from 16 days after MMLV-GFP injection and their density rapidly increases until 56 dpi. Spine density stabilizes at 56 dpi, although specific subtypes of spines continue to develop until 126 dpi (ref. 36). nGCs receive functional inputs from the entorhinal cortex, septum and hippocampal subfields^37^. Therefore, we determined nGC spine density in the inner-molecular layer (IML) and the outer-molecular layer (OML) where afferent inputs contact nGCs (refs 38, 39) (Fig. 4g,h). In line with previous work, wild-type nGCs exhibited an average spine density of ±1.6 spines μm^−1^ at 56 dpi of MMLV CAG-GFP (Fig. 4i) (ref. 39). Loss of Sema7A resulted in a mild but consistent decrease in spine density on GFP-labelled nGCs in the IML. Although spine density appeared to be also reduced in the OML of *Sema7A* mutants, this effect was not statistically significant (Fig. 4i; *n*=5 mice*, post-hoc t*-test: *P*_(OML)_=0.0812 and *P*_(IML)_=0.0320). Quantification of mushroom-like spine density, a hallmark of matured spines^40^,^41^, in both the IML and OML revealed reduced mushroom-like spine density in the IML of *Sema7A*^−/−^ nGCs (*n*=5 mice, Student's *t*-test: *Sema7A*^*+/+*^_(IML)_=0.200±0.011, *Sema7A*^*−/−*^_(IML)_=0.160±0.010, *P*=0.033, *Sema7A*^*+/+*^_(OML)_=0.200±0.021, *Sema7A*^*−/−*^_(OML)_=0.170±0.017, *P*=0.674 (mean±s.e.m.)). At 80 dpi, spine density was still reduced in the IML of *Sema7A* mutants and was also significantly decreased in the OML (Fig. 4j; *n*=3 mice, two-way ANOVA IML: genotype F_(1, 12)_=15.094, *P*=0.0022, OML: genotype F_(1,12)_=10.57, *P*=0.0069, *post-hoc t*-test: *P*_(IML)_=0.0458 *P*_(OML)_=0.0463). Thus, Sema7A positively regulates dendritic spine density in nGCs.

### Sema7A is required for functional integration of nGCs

**Figure 5:**
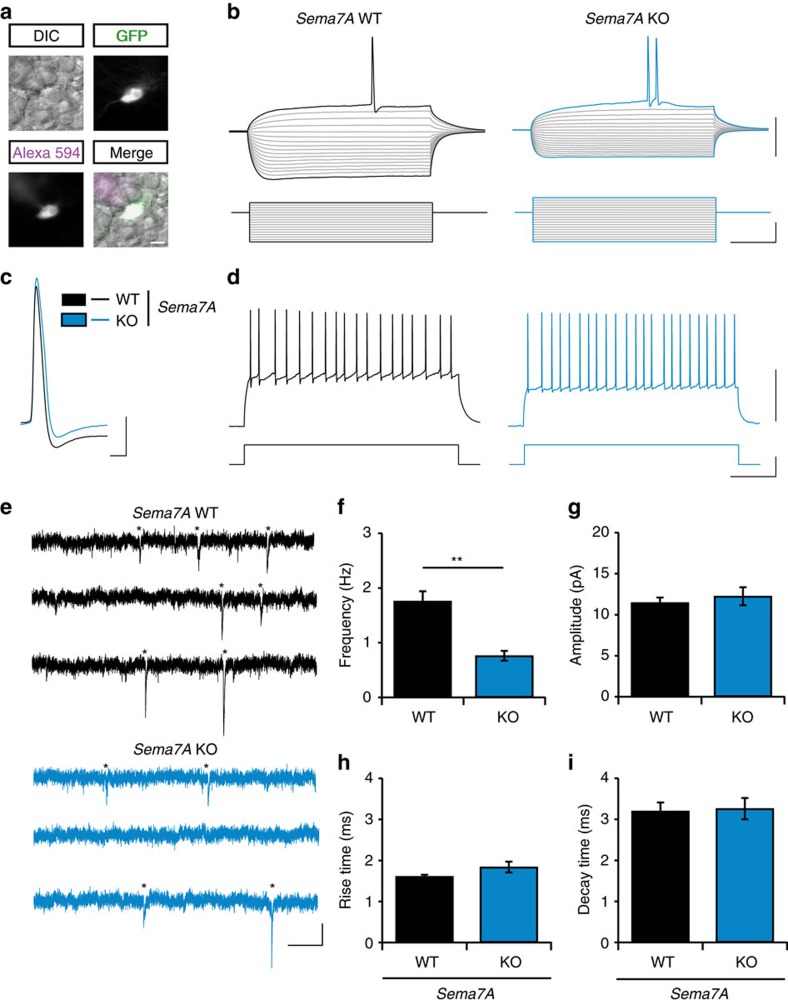
Sema7A is required for the functional integration of adult-born DG granule cells. (**a**) Representative images of an MMLV CAG-GFP infected adult-born granule cell (nGC) used for recording of electrophysiological properties and miniature excitatory post-synaptic currents (mEPSCs). Healthy cells were identified using differential interference contrast (DIC). Recording of nGCs (GFP-positive cells) was confirmed by the presence of recording pipet-dye Alexa Fluor 594 in the same cell. (**b**) Representative traces of membrane potential (upper traces) in response to current injections (lower traces) in *Sema7A*^*+/+*^ (WT, black) or *Sema7A*^*−/−*^ (KO, blue) nGCs. Note the reduced hyperpolarization of the membrane potential during hyperpolarizing current injections in KO nGCs. (**c**) Representative traces of action potentials in WT and KO nGCs. (**d**) Representative traces for action potential firing rates (upper trace) in response to a 200 pA current injection (lower trace) in WT and KO nGCs. (**e**) Representative traces of mEPSC recordings in WT and KO nGCs. Asterisks indicate identified mEPSC events. (**f**–**i**) Quantification of mEPSC instantaneous frequency (**f**), amplitude (**g**), rise- (**h**) and decay- (**i**) time (*n*_(WT)_=9 cells/3 mice, *n*_(KO)_=8 cells/3 mice, Student's *t*-test). Data are presented as means±s.e.m. **P<0.01. Scale bars: (**a**) 10 μm, (**b**,**d**) horizontal: 220 ms, vertical: 50 mV (upper traces) or 200 pA (bottom traces), (**c**) horizontal: 2 ms, vertical: 20 mV, (**e**) horizontal: 200 ms, vertical: 10 pA.

The hampered dendritic development and spinogenesis of nGCs observed in *Sema7A*^*−/−*^ mice may affect the functional integration of nGCs into the adult DG. To test this hypothesis, we performed an electrophysiological characterization of nGCs in the DG at 56 days after MMLV-GFP injection (Fig. 5a–d, Supplementary Table 1). Normal resting membrane potential, action potential membrane threshold and action potential properties were recorded in nGCs of *Sema7A*^*−/−*^ mice (Supplementary Table 1). In *Sema7A*^*−/*−^ mice, nGCs had lower input resistance and required higher current injections to reach the action potential threshold (Supplementary Table 1; *n*=3 mice, Student's *t*-test with Benjamini-Hochberg correction for multiple testing: *P*_(input resistance)_=0.007, *P*_(hyperpolarization during −300 pA injection)_=0.001, *P*_(action potential current threshold)_=0.017). To test whether decreased dendritic development and spinogenesis resulted in reduced functional inputs onto nGCs in *Sema7A*^*−/−*^ mice, we next recorded miniature excitatory post-synaptic currents (mEPSCs) (Fig. 5e–i). Quantification of mEPSCs showed normal amplitudes, rise- and decay times (Fig. 5g–i; *n*=3 mice, Student's *t*-test: *P*_(amplitude)_=0.553, *P*_(rise time)_=0.112, *P*_(decay time)_=0.855). However, the instantaneous frequency of the recorded mEPSCs was reduced in *Sema7A*^*−/−*^ mice nGCs (Fig. 5f; Student's *t*-test: *P*<0.001). To test whether Sema7A is also required for the function of adult GCs, we determined electrophysiological properties of putative mature GCs (GFP-negative GCs in the outer layer of the DG GCL). Normal electrophysiological properties and excitatory synaptic inputs were observed in adult GCs of *Sema7A*^*−/−*^ mice (Supplementary Fig. 9, Supplementary Table 2). Overall these observations suggest that Sema7A, as expected on basis of delayed dendritic development and reduced (mature) spine density, is required for the proper functional integration of nGCs and their excitatory inputs.

### Stage-specific receptor interactions control neurogenesis

**Figure 6:**
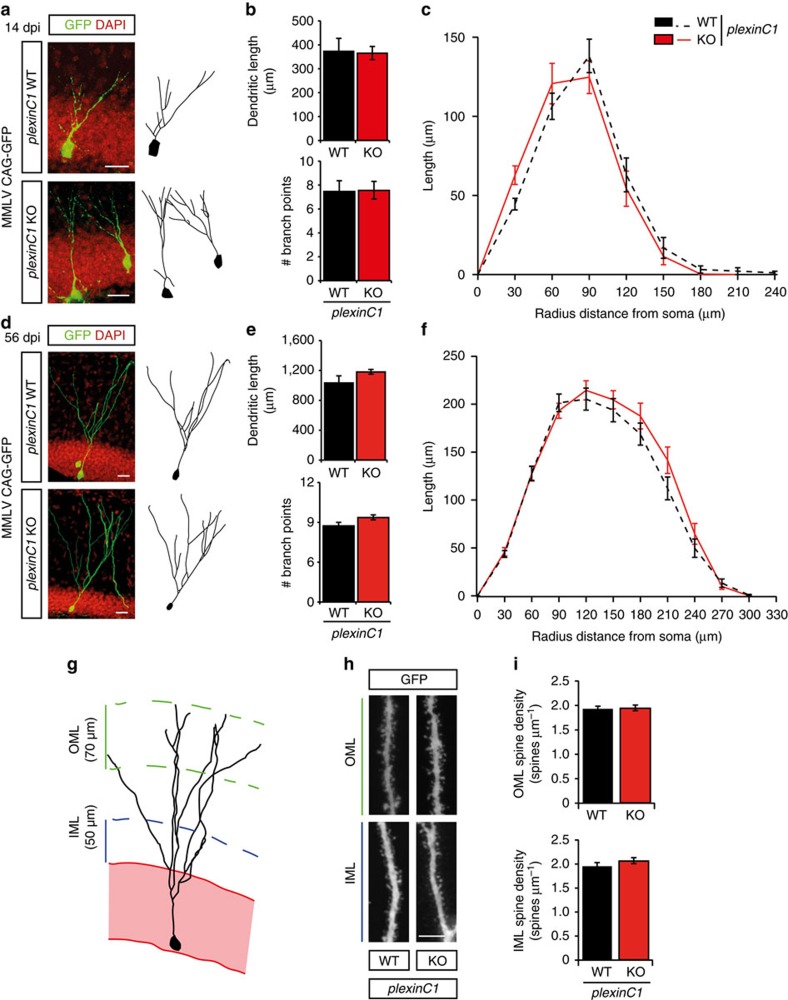
Dendritic growth and spine development are intact in adult-born DG granule cells in *plexinC1*^*−/−*^ mice. (**a**,**d**) The dentate gyrus (DG) of adult *plexinC1*^*+/+*^ (WT) or *plexinC1*^*−/−*^ (KO) mice was injected with MMLV CAG-GFP to label adult-born granule cells and immunostained for GFP at 14 or 56 days post-injection (dpi). DAPI is shown in red. Right panels depict representative tracings based on confocal z-stack images. (**b**,**e**) Quantification of dendritic length and number of branch points in WT (black bars) and KO (red bars) littermates (two-way ANOVA, *post-hoc t*-test, 14 dpi: *n*=4, *P*_(length)_=0.898 and *P*_(branch points)_=0.944, 56 dpi: *n*=5, *P*_(length)_=0.344 and *P*_(branch points)_=0.159). (**c**,**f**) Sholl-analysis of the dendritic tree of GFP-labelled cells in WT (black dashed-lines) and KO mice (red lines). Dendritic length per radius distance from soma (30 μm radius interval) was unchanged in KO mice (14 dpi: *n*=45 WT cells/4 mice, *n*=47 KO cells/4 mice, 56 dpi: *n*=40 WT cells/5 mice, *n*=38 KO cells/5 mice. Repeated-measures ANOVA 14 dpi: F_(7, 630)_=1.833, *P*=0.139, 56 dpi: F_(9, 684)_=1.075, *P*=0.364). (**g**) Schematic representation of the DG indicating the regions selected for spine density quantification. IML, inner molecular layer; OML, outer molecular layer. (**h**) Representative images showing spine-bearing dendritic segments in the OML and the IML in WT and KO mice. (**i**) Quantification of spine density on adult-born granule cells at 56 dpi did not reveal differences between WT (*n*=5) and KO (*n*=5) mice (Student's *t*-test, *P*_(OML)_=0.8858 and *P*_(IML)_=0.2825). Whiskers represent s.e.m. Scale bars: (**a**,**d**) 25 μm; (**h**) 10 μm.

Our data indicate that Sema7A controls cell proliferation in the SGZ of the DG through the plexinC1 receptor. However, given the restricted expression of plexinC1 in early progenitors in the adult DG (Fig. 1, Supplementary Fig. 2), it seemed unlikely that this receptor could also mediate regulation of dendritic outgrowth and spine development by Sema7A in nGCs. Indeed, injection of adult *plexinC1*^*−/−*^ mice with MMLV CAG-GFP followed by tracing of the dendritic tree of nGCs at 14 dpi and 56 dpi did not reveal defects in cell migration or dendritic outgrowth and ramification (Fig. 6a–f, Supplementary Fig. 8d,e,h,i). Furthermore, spine densities of nGC dendrites within the IML and OML of adult *plexinC1*^*−/−*^ mice were reminiscent of those found in wild-type littermates (Fig. 6g–i).

**Figure 7:**
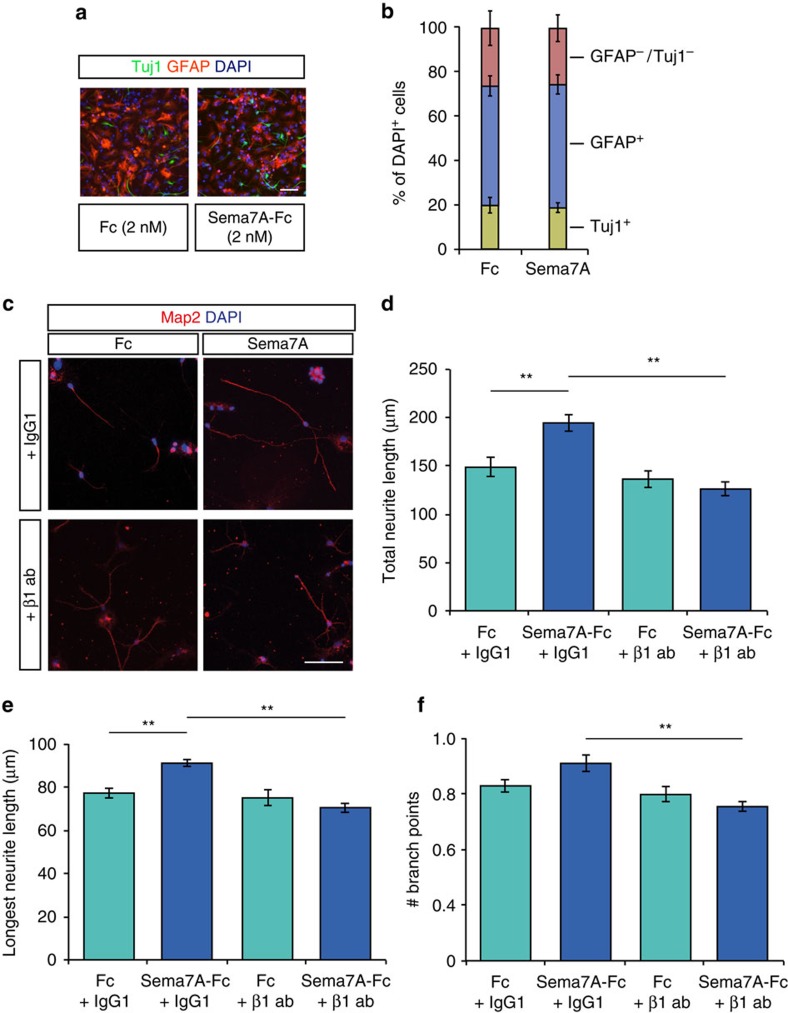
Sema7A promotes neurite growth from differentiated adult SGZ neuronal progenitors *in vitro*. (**a**) Immunocytochemistry for Tuj1 and glial fibrillary acidic protein (GFAP) on cultures of differentiated SGZ neuronal progenitors at 3 days post-differentiation in the presence of 2 nM Fc control or Sema7A-Fc protein. (**b**) Quantification of the number of GFAP-negative/Tuj1-negative, GFAP-positive (astrocytes) or Tuj1-positive (neurons) cells in cultures of differentiated SGZ neuronal progenitors. Addition of Sema7A-Fc does not alter the relative number of neurons and astrocytes in the cultures. (**c**) Immunocytochemistry for microtubule-associated protein 2 (Map2) to label dendrites on cultures of differentiated SGZ neuronal progenitors at 10 days post-differentiation in the presence of 2 nM exogenous Fc control or Sema7A-Fc protein in the presence of a β1-integrin blocking antibody or control IgG1 antibody. (**d**–**f**) Quantification of total Map2^+^ neurite length (**d**), longest Map2^+^ neurite length (**e**) or the number of Map2^+^ branch points (**f**) in cultures as in **c** (two-way ANOVA, *post-hoc* Replicate-paired *t*-test). Data represent means±s.e.m. from at least thre independent experiments. *P<0.05 and **P<0.01. Scale bars: 50 μm.

**Figure 8:**
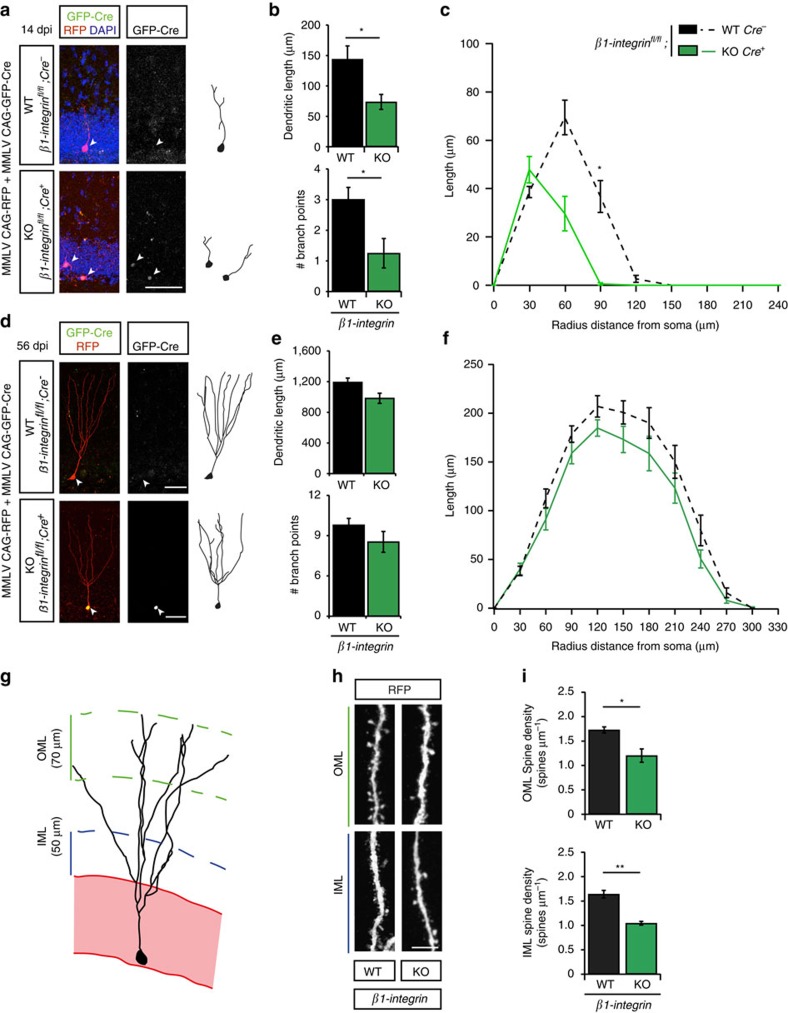
β1-integrins are required for dendritic growth and spine development in adult-born DG granule cells. (**a**,**d**) The dentate gyrus of *β1-integrin*^*fl/fl*^ mice was injected with MMLV CAG-RFP and MMLV CAG-GFP-Cre and immunostained for RFP and GFP at 14 or 56 days post-injection (dpi). Despite underestimation of the dendritic length and complexity due to low expression of RFP at 14 dpi, cell-autonomous ablation of *β1-integrin* in GFP-positive, RFP-positive cells induces a clear reduction in dendritic morphology at 14 dpi. DAPI is shown in blue. WT, GFP-negative, RFP-positive cell; KO, GFP-positive, RFP-positive cell. Right panels depict representative tracings based on confocal z-stack images. (**b**,**e**) Quantification of dendritic length and number of branch points in WT (black bars, *n*=4) and KO (green bars, *n*=4) littermates (two-way ANOVA, *post-hoc t*-test, 56 dpi: *P*=0.176 and *P*=0.336). (**c**,**f**) Sholl-analysis of the dendritic tree of WT (black dashed-lines) and KO cells (green lines). Dendritic length per radius distance from soma (30 μm radius interval) was decreased in KO adult-born granule cells (nGCs) (14 dpi: *n*=20 WT cells/4 mice, *n*=9 KO cells/4 mice, 56 dpi: *n*=27 WT cells/4 mice, *n*=20 KO cells/4 mice. Repeated-measures ANOVA 14 dpi: F_(7, 175)_=2.625, 56 dpi: F_(9, 405)_=0.724, *P*=0.533). (**g**) Schematic representation of the DG indicating the regions selected for spine density quantification. IML, inner molecular layer; OML, outer molecular layer. (**h**) Representative images showing spine-bearing dendritic segments in the OML and the IML in WT and KO nGCs. (**i**) Quantification of spine density on nGCs at 56 dpi reveals differences between WT (*n*=4) and KO (*n*=4) granule cells (two-way ANOVA, *post-hoc t*-test). Data are represented as means±s.e.m. *P<0.05 and **P<0.01. Scale bars: (**a**,**d**) 25 μm; (**h**) 10 μm.

To determine whether β1-integrin receptors are involved in Sema7A-mediated dendritic outgrowth in nGCs, we first differentiated aNPCs *in vitro* and measured neurite growth and branching. Importantly, differentiation of cultured aNPCs in the presence of Sema7A-Fc (2 nM) for 3 days did not alter the percentage of GFAP^+^ or Tuj1^+^ cells compared to control treatment (Fig. 7a,b; *n*=3 independent experiments, Chi-squared test: *P*=0.3008, *X*^2^=2.4024). This observation is in line with our finding that the number of NeuN^+^/BrdU^+^ cells is unchanged in *Sema7A*^*−/−*^ mice *in vivo* (Fig. 3). To confirm that Sema7A stimulates neurite outgrowth of differentiated aNPCs, we quantified the length of Map2^+^ neurites at DIV10 (Fig. 7c–e). In line with a reduction in dendrite growth in *Sema7A*^*−/−*^ mice *in vivo* (Fig. 4), exposure to Sema7A (2 nM) significantly enhanced total neurite length and longest neurite length *in vitro* (Fig. 7c–e; *n*=3 independent experiments, *post-hoc t*-test: *P*_(total)_=0.008 and *P*_(longest)_=0.007). Functional blockage of β1-integrins by incubation with a well-characterized β1-integrin blocking antibody^5^ prevented Sema7A's positive effect on neurite outgrowth (Fig. 7c–e, *n*=3 independent experiments, two-way ANOVA total: protein × antibody treatment F_(1, 8)_=13.353, *P*=0.006, longest: protein × antibody treatment F_(1, 8)_=10.515, *P*=0.012, *post-hoc t*-test: *P*_(total)_=0.002 and *P*_(longest)_=0.001). Analysis of neurite ramification was hindered by the relatively small number of branch points per neurite on differentiated nGCs even at DIV 10 (Fig. 7f). While no statistical significant effect of Sema7A-Fc on number of branch points was observed, β1-integrin blocking antibody treatment reduced the number of branch points on neurites compared to Sema7A-Fc treated cells (Fig. 7f; *n*=3 independent experiments, two-way ANOVA branch points: protein × antibody treatment F_(1, 8)_=6.077, *P*=0.039*, post-hoc t*-test: *P*=0.001). In all, these data show that Sema7A is not required for neuronal differentiation but that it stimulates dendrite outgrowth of differentiated nGCs via β1-integrins. To confirm this model a mix of retroviral MMLV CAG-GFP/Cre and MMLV CAG-RFP viruses was injected in adult *β1-integrin*^*fl/fl*^ mice. The RFP signal in nGCs allows for tracing and analysis of dendritic morphology and spine density, whereas co-expression of the Cre-vector, reported by nuclear GFP immunostaining, induces cell-autonomous ablation of *β1-integrin* in individual nGCs (ref. 35). Similar to *Sema7A*^*−/−*^ mice (Fig. 4), cell-autonomous loss of *β1-integrin* in nGCs induced a reduction in dendritic outgrowth and complexity at 14 dpi (Fig. 8a–c; *n*=4 mice, two-way ANOVA length: genotype F_(1,12)_=9.738, *P*=0.0088, branch points: genotype F_(1,12)_=6.205, *P*=0.0284 *post-hoc t*-test: *P*_(dendritic length)_=0.0307 and *P*_(branch points)_=0.0292, Supplementary Fig. 10a; *n*=4 mice, repeated-measures ANOVA: Sholl length; F_(7, 175)_=2.625, *P*=0.013, Sholl intersections; F_(7, 175)_=5.273, *P*<0.001). Unilateral injection of MMLV CAG-GFP-IRES-Cre or MMLV CAG-GFP in a separate *β1-integrin*^*fl/fl*^ cohort confirmed that the observed defects were not caused by a potential increased cellular burden due to infection with two viral constructs (Supplementary Fig. 10c,d; *n*=4 mice, Student's *t*-test: *P*_(length)_*=*0.0445 and *P*_(branch points)_=0.0254). Intriguingly, the temporally restricted requirement for Sema7A during early stages (14 dpi, Fig. 4a–c) of dendritic outgrowth but not at later stages (56 dpi, Fig. 4d–f) was also observed in virus-injected *β1-integrin*^*fl/fl*^ mice. No significant change in total dendritic length, number of branch points or dendritic complexity, was observed at 56 dpi in *β1-integrin*^*−/−*^ nGCs (Fig. 8d–f, Supplementary Fig. 10b). Thus, β1-integrins are required at early stages of dendritic outgrowth in nGCs.

Loss of Sema7A, but not plexinC1, caused reduced spine density in nGCs (Fig. 4). To determine whether or not β1-integrin knockout induces similar defects, spine densities were analysed within the IML and OML of *β1-integrin*^*−/−*^ nGCs at 56 dpi (Fig. 8g,h). Cell-autonomous ablation of β1-integrin in nGCs resulted in a marked reduction of spine density both in the IML and OML (Fig. 8h,i; *n*=4 mice, two-way ANOVA genotype F_(1,12__=37.235, *P*<0.0001 *post-hoc t*-tests: *P*_(OML)_=0.012 and *P*_(IML)_=0.002). A significant reduction in mushroom-like spine density was observed in the OML but not in the IML (*n*=5 mice, Student's *t*-test: *β1-integrin*^*+/+*^_(IML)_=0.248±0.029, *β1-integrin*^*−/−*^_(IML)_=0.211±0.020, *P*=0.341, *β1-integrin*^*+/+*^_(OML)_=0.319±0.026, *β1-integrin*^*−/−*^_(OML)_=0.205±0.017, *P*=0.011 (mean±s.e.m.)). These data together with our *in vitro* experiments (Fig. 7) suggest that Sema7A-β1-integrin signalling controls dendritic development and spine density of nGCs within the adult DG.

## Discussion

Sema7A contributes to the formation of neuronal connections during development. Although neural expression of Sema7A increases as development progresses, the precise role of this semaphorin in the adult brain remains poorly understood. Here, we report several stage-specific roles for Sema7A during adult hippocampal neurogenesis. At early stages, Sema7A and its receptor plexinC1 cooperate to suppress early progenitor cell proliferation in the adult SGZ. At later stages, Sema7A regulates dendrite growth, complexity and spine development of adult-born GCs. These morphological effects require β1-subunit-containing integrin receptors but not plexinC1. In addition, loss of Sema7A results in reduced excitatory inputs to nGCs but not to adult GCs. These data support a functional role for Sema7A signalling during adult neurogenesis. Our data establish a novel role for Sema7A and plexinC1 in the adult brain and, more generally, implicate semaphorins and plexins in the control of cell proliferation during adult hippocampal neurogenesis. Moreover, they provide further insight into the function and mechanisms of β1-integrins during adult neurogenesis. Recent work shows that regulation of neuroglial plasticity at the adult median eminence by Sema7A also requires plexinC1 and β1-integrins^11^. Our data are, however, conceptually distinct from these previous observations: here we find that stage-specific neuronal expression of Sema7A receptors is required for adult neurogenesis, rather than a requirement for receptor expression in axons (plexinC1) and ensheating glia (β1-integrins). Further, while it is known that semaphorins can regulate multiple, distinct steps of neuronal development^1^, our data present one of the first examples in which stage-specific expression of structurally unrelated semaphorin receptor proteins (that is, plexins and integrins) in the same neuron is required to mediate these steps.

**Figure 9:**
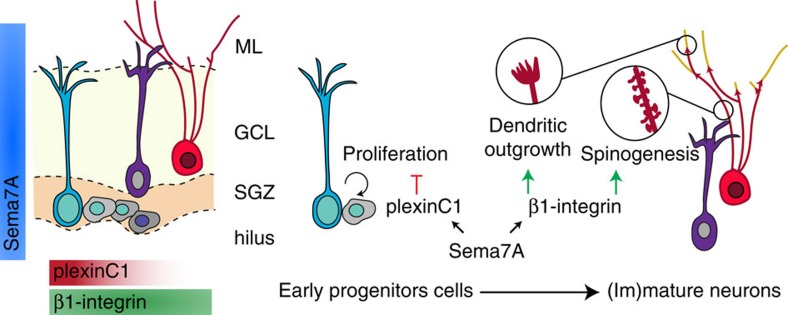
Sema7A regulates cell proliferation and dendritic morphology during adult hippocampal neurogenesis via distinct receptors. In the adult dentate gyrus (DG), expression of plexinC1 is largely confined to early progenitor cells in the subgranular zone (SGZ). In contrast, Sema7A and β1-subunit-containing integrin receptors are expressed throughout the SGZ and granular cell layer (GCL). On basis of our *in vitro* and *in vivo* results we propose a model in which Sema7A, through plexinC1, inhibits proliferation of early progenitor cells in the SGZ during early stages of adult hippocampal neurogenesis, while at later stages Sema7A utilizes β1-subunit-containing integrin receptors to promote dendritic growth, complexity and spine development in differentiated adult-born GCs. While our data suggest that β1-subunit-containing integrin receptors are required cell autonomously in adult-born GCs, further work is needed to determine the cellular origin of the Sema7A that activates these receptors. Sema7A could be expressed in nGCs and activate β1-subunit-containing integrin receptors in *cis*. Alternatively, Sema7A may be expressed in neighbouring nGCs and act in *trans* either via cell-cell interactions or following cleavage of its GPI domain, resulting in a soluble Sema7A ectodomain. Similarly, the cellular origin of Sema7A molecules that activate plexinC1 receptors remains to be determined. Sema7A is broadly expressed in the GC layer, including strongly in immature and mature granule cells. These cells could represent a cellular source of Sema7A for aNPCs in the adjacent SGZ.

Integration of nGCs in mature hippocampal circuitry constitutes an important form of structural plasticity that contributes to hippocampal function and, if perturbed, to disease^20^,^42^,^43^,^44^. Most aNPCs in the adult DG are maintained in a quiescent state to secure tissue homeostasis and avoid stem cell exhaustion, with proliferation induction occurring. The neurogenic niche is composed of various cell types and molecular signals that tightly control aNPC quiescence and proliferation^21^,^22^. Our data identify Sema7A and plexinC1 as new players in the regulation of aNPC proliferation in the adult DG and suggest that Sema7A acts through plexinC1 to inhibit aNPC proliferation in the SGZ (Fig. 9). Strong *plexinC1* expression has recently been detected in quiescent, as compared to active aNPCs in the SVZ (ref. 23). Our data suggest that plexinC1 protein is present in both quiescent and active aNPCs. This indicates that plexinC1 may act to retain aNPCs in a quiescent state but may also serve other functions, for example to negatively control transiently amplifying cells, or IPCs. In contrast to plexinC1, which is largely confined to early progenitor cells, Sema7A is expressed in both aNPCs and their immediate environment. The observation that application of exogenous Sema7A inhibits neurosphere formation *in vitro* while genetic ablation of Sema7A has no effect hints at a non-cell autonomous role for Sema7A in aNPC proliferation. Sema7A is broadly expressed in the GC layer, including strongly in immature and mature GCs. These cells could represent a cellular source of Sema7A for aNPCs in the adjacent SGZ. Additional studies will examine these possible non-cell autonomous effects and dissect the precise plexinC1 mechanism-of-action in the SGZ.

Regulation of cell proliferation by plexinC1 is likely not restricted to the SGZ. PlexinC1 is prominently expressed in different neurogenic niches in the embryonic and adult mouse brain^3^,^23^ (Supplementary Fig. 1a–f). Further, plexinC1 is a tumour suppressor for melanoma. Loss of plexinC1 increases melanocyte proliferation thereby promoting melanoma progression^18^. Interestingly, another plexin family member, plexinB2, is expressed in adult progenitor cells in the SVZ. PlexinB2 promotes cell proliferation in the adult SVZ and neuroblast migration in the RMS and olfactory bulb^45^. These results coupled with our data detailing a role for plexinC1 in adult hippocampal neurogenesis reveal that plexins can have opposing effects on aNPC proliferation and highlight a more general role for these receptors in adult neurogenesis. Plexins other than plexinB2 and plexinC1 regulate cell proliferation in normal and diseased tissues^46^,^47^,^48^,^49^, and it will be interesting to determine whether or not these receptors contribute to adult neurogenesis.

The best-characterized neuronal receptors for Sema7A are β1-subunit-containing integrins. β1-integrins transduce Sema7A-mediated effects on axonal and dendritic growth, neuron migration and synapse elimination^5^,^6^,^9^,^10^,^11^. β1-integrins contribute to adult neurogenesis and their selective inactivation affects cell proliferation, neuroblast migration and neuronal differentiation in the adult DG or SVZ/RMS (refs 29, 30, 50). In the postnatal cerebellum, plexinC1 and β1-integrins cooperate to mediate the synaptic effects of Sema7A (ref. 6). Therefore, we examined whether, in addition to plexinC1, β1-integrins regulate aNPC proliferation. We found that cell proliferation in the SGZ is intact in adult *β1-integrin*^*fl/fl*^;*nestin-Cre* mice at 2 h and 3 days following BrdU labelling. Thus, while the two known Sema7A receptors plexinC1 and β1-integrins are both expressed in aNPCs, our data suggest that plexinC1 is required for mediating the proliferation suppressive effects of Sema7A. First, plexinC1 and Sema7A mutant mice, but not *β1-integrin*^*fl/fl*^;*nestin-Cre* mice, display similar changes in the number of proliferating cells in the SGZ using different approaches. Second, a negative effect of Sema7A on neurosphere growth is seen in wild-type but not *plexinC1*^*−/−*^ neurospheres. Third, neurospheres exposed to Sema7A show increased phosphorylation of cofilin, but not of β1-integrin receptors (Supplementary Figs 3d–g and 4). Normally activation of plexinC1 by Sema7A leads to phosphorylation of cofilin, while binding of Sema7A to β1-integrins triggers their phosphorylation^11^,^12^. Thus, Sema7A activates cofilin but not β1-integrins in neurosphere cultures. Together our observations indicate that the interaction of Sema7A with plexinC1, but not with β1-subunit containing integrin receptors, triggers downstream signalling during Sema7A-induced suppression of aNPC proliferation. This is in line with previous work showing normal cell proliferation in the SVZ of adult *β1-integrin*^*fl/fl*^;*nestin-Cre* mice^50^. It should be noted, however, that acute inactivation of β1-integrins in the SVZ by *in vivo* injection of function blocking antibodies causes an increase in aNPC proliferation^30^. One explanation for these seemingly disparate results is that compensatory mechanisms mediated by other classes of integrins, for example, β8-integrins^51^, or other molecules in the neurogenic niche mask putative effects of genetic β1-integrin ablation on aNPC proliferation. Thus, while our data implicate plexinC1 in Sema7A's inhibitory effects on aNPC proliferation, further work is needed to examine the role of β1-integrins and to understand how Sema7A signals through plexinC1, but not β1-integrins, in aNPCs despite the presence of both receptors in these cells.

A large body of previous work has identified key factors for the regulation of aNPC proliferation in the SGZ, but the extracellular molecular signals that control the morphological development of adult-born GCs are less well-understood (for example, refs 52, 53, 54, 55, 56). Our data help to fill this void by showing that Sema7A is indispensable for the early development of dendrites and dendritic spines in adult-born GCs and that this effect cell-autonomously requires β1-integrins but not plexinC1. Interestingly, we find that genetic ablation of β1-integrins leads to a larger decrease in dendritic length and spine number as compared to knockout of Sema7A. This suggests that different β1-integrin ligands may cooperate to control dendritic morphology during adult hippocampus neurogenesis. Multiple different β1-integrin ligands have been implicated in adult neurogenesis^57^. Recent work further indicates that, in contrast to Sema7A, Sema5A acts to inhibit spine density and synaptic integration of nGCs via plexinA2 (ref. 55). This suggests that semaphorins and their receptors can exert opposing effects on developing spines during the integration of nGCs into the adult DG.

All steps of adult hippocampal neurogenesis occur in the SGZ and DG in relative close proximity and many of the factors regulating multiple distinct steps, such as Sema7A, are expressed constantly. Intriguingly, our study, and work of others^56^ reveals that these non-selectively expressed cues can exert stage-specific effects because of the stage-dependent expression and function of their receptors: plexinC1 expression is restricted to aNPCs and regulates the effects of Sema7A on proliferation, while β1-integrins serve as a Sema7A receptor in differentiated adult-born GCs. Stage-specific receptor expression not only allows spatiotemporal control over the biological effects of Sema7A during adult neurogenesis, but it also diversifies the effects of this semaphorin, enabling it to exert control over proliferation and morphogenesis. In contrast to GCs generated at embryonic stages, adult-born GCs integrate into a fully developed neural circuit. However, many of the molecular cues that help to establish neural circuitry during development are no longer present in the adult brain. During embryonic development, the effects of semaphorins, and other cues, are diversified allowing them to control a disproportionally large number of cells and cellular events^58^,^59^,^60^,^61^,^62^,^63^,^64^,^65^. Whether similar diversification mechanisms operate in the adult CNS and, if so, whether these would contribute to the regulation of adult neurogenesis was not known. Our data suggest that diversification mechanisms for semaphorins operate in the adult brain and help to explain how a relatively limited set of molecular cues can spatiotemporally control the birth and integration of adult-born GCs. It will be interesting to determine whether similar mechanisms act to control other roles of semaphorins in the adult brain, such as in synaptic function^66^.

In summary, the data presented here show novel stage-specific effects of Sema7A during adult hippocampal neurogenesis. Remarkably, these effects, including suppression of aNPC proliferation and promotion of dendritic development, require stage-specific expression of structurally unrelated Sema7A receptors (plexinC1 and β1-integrins). This tight regulation of receptor expression not only provides spatiotemporal control over Sema7A's effects during adult neurogenesis, but also diversifies the biological effects of this semaphorin.

## Methods

### Animals

All experiments were approved by the DEC (Dutch Experimental Review Committee), performed in line with institutional guidelines of the University Medical Center Utrecht and Vrije University Amsterdam, and were conducted in agreement with Dutch law (Wet op de Dierproeven, 1996) and European regulation (Guideline 86/609/EEC). C57BL/6J mice were obtained from Jackson Laboratories. *β1-integrin*^*fl/fl*^ and *nestin-Cre* mice were obtained from Jackson Laboratories. *Sema7A*^*−/−*^ and *plexinC1*^*−/−*^ mice were as described previously^5^. All mouse lines used in this study were backcrossed at least five times into a C57BL/6J genetic background. *β1-integrin*^*fl/fl*^*;nestin-Cre*^*+/−*^ mice were obtained by paternal inheritance of *nestin-Cre*^*+/−*^ to ensure efficient Cre recombination. All mice were raised by their mothers and weaned at the age of 4 weeks. Mice were kept in a controlled 12 h light-dark cycle with a temperature of 22±1 °C and were given unrestricted access to food (211 RMH-TM diet; Hope Farms) and water. Mice were housed in transparent plexiglas cages with wood-chip bedding and paper tissue for nest building.

### BrdU-pulse labelling

BrdU-pulse labelling was performed as described previously^27^. For the analysis of cell proliferation in the adult SGZ, 8-week-old *plexinC1*^*−/−*^, *Sema7A*^*−/−*^*, β1-integrin*^*fl/fl*^*;nestin-Cre*^*+/−*^ and littermate control mice received intraperitoneal injections of 100 mg kg^−1^ BrdU in saline. The 2 h post-injection groups received one injection of BrdU. The 3 days and 28 days post-injection (dpi) groups received two injections of BrdU separated by an 8 h interval. For Supplementary Fig. 6h, 6 and 18-month-old *plexinC1*^*−/−*^ and littermate control mice received two injections of BrdU and were treated identical to the 3 dpi group.

### Viral vector production

Viral vector production was as described previously^35^,^37^. MMLV retrovirus was produced by transient transfection of CAG-GFP (Addgene plasmid #16664), CAG-GFP/Cre (Addgene plasmid #49054, encoding for GFP fused Cre recombinase; both were kind gifts of Fred Gage (Salk Institute for Biological Studies))^36^, CAG-RFP, or CAG-GFP-IRES-Cre (Addgene plasmid #48201; 2.5 μg, encoding for GFP and Cre recombinase separately) with CMVGagPol (5 μg) and CMV-VSVG (2.5 μg) vectors using Lipofectamine 2000 in 90% confluent 293T cells^34^. Virus-containing supernatant was harvested 36 h after transfection. Supernatant was collected, filtered and concentrated by ultracentrifugation (two times 2 h at 19,400 r.p.m.). Viral titres were estimated to be ∼10^8^ i.u. (infectious units) ml^−1^ as determined by infection of serially diluted virus in 293T cells.

### Stereotaxic injections

Stereotaxic injections were performed as described previously^35^,^37^. Mice received pre-, peri- and post-surgically analgesia (5 mg kg^−1^ Carprofen, subcutaneous injection; Lidocaine on the skull) and were anaesthetized using Isoflurane (4% induction, 1–2% maintenance). Stereotaxic surgery was performed to deliver 1.0 μl of retrovirus (10^4^–10^5^ i.u. μl^−1^, MMLV) bilaterally to the DG using the following spatial coordinates relative to bregma: AP=−2.1 mm, lateral=1.5 mm/−1.5 mm and ventral=−2.4 mm. For some retroviral injections in *β1-integrin*^*fl/fl*^ mice a mixture (1:1) of MMLV CAG-RFP and MMLV CAG-GFP/Cre or MMLV CAG-GFP-IRES-Cre was used.

### Tissue collection and sectioning

For immunohistochemistry, adult mice (>8 weeks) were deeply anesthetized with Euthanimal (Alfasan) and intracardially perfused with ice-cold phosphate-buffered saline (PBS) followed by 4% paraformaldehyde (PFA) in PBS. After dissection, brains were incubated overnight (ON) in 4% PFA in PBS at 4 °C. PFA-fixed brains were then incubated in 30% sucrose in PBS until saturation, snap-frozen in −20 °C isopentane and stored at −80 °C. For non-radioactive *in situ* hybridization, mice were killed by cervical dislocation. Dissected brains were directly frozen on dry ice and stored at −80 °C. Frozen brains for expression analysis and BrdU-pulse assays were sectioned on a cryostat (Leica CM3050). For the BrdU-pulse assay, 40 μm coronal brain sections were collected in cryoprotectant solution and stored at −20 °C until use for free-floating immunohistochemistry. For immunohistochemistry and non-radioactive *in situ* hybridization expression analysis, coronal sections (16–20 μm) containing the hippocampus were collected on Superfrost slides (Merzel, Germany). Slides were stored at −20 °C until use for immunohistochemistry and −80 °C for non-radioactive *in situ* hybridization. Coronal sections (100 μm) of brains injected with MMLV were generated on a vibratome (VT1000S Leica), and stored in cryoprotectant solution, containing 40% PBS, 30% glycerol, 30% ethyleneglycol, at −20 °C until being subjected to free-floating immunohistochemistry.

### Immunohistochemistry

Immunohistochemistry was performed as described^67^. In brief, all sections were blocked for 1 h in PBS containing 5% normal donkey serum or normal goat serum and 0.5–1.0% Triton X-100. Prior blocking, BrdU immunohistochemistry required antigen-retrieval. Sections were incubated in 2* *N HCl at 37 °C for 30 min followed by 10 min incubation in 0.1 M boric acid, pH 8.0. Then slices were treated with 0.3% peroxide in PBS to block endogenous peroxidase activity. Following incubation in blocking solution, all sections or coverslips were incubated overnight with primary antibodies in blocking solution at 4 °C. The following day, sections were incubated with appropriate secondary antibodies in blocking buffer for 2 h at room temperature (RT) (Alexa Fluor 488, 555, 568 or 596, 647, or biotin-conjugated (for BrdU staining)). Extensive washing in PBS was performed in between every incubation step. For BrdU immunohistochemistry, sections were incubated in ABC-staining solution (Pierce) for 2 h at RT, and developed with DAB (Sigma-Aldrich) in 0.05 M Tris-buffer, pH 7.6. Immunofluorescently stained sections were counterstained with DAPI (Sigma-Aldrich) and mounted in DAPCO (Sigma-Aldrich) or Prolong Gold anti-fade reagent (Invitrogen). For plexinC1 immunohistochemistry antigen retrieval was required. Sections were incubated in 10 mM sodium citrate (pH 8.5) for 7 min in the microwave at 180 W followed by 2 h incubation in blocking solution. Sections were blocked for 2 h and incubated over night at 4 °C with primary antibodies. Incubation with secondary antibodies conjugated to biotin was followed by incubation with streptavidin-conjugated Alexa Fluor-488 for 2 h at RT. For differentiation assay immunocytochemistry, coverslips were fixed with 8% PFA and blocked for 1 h. Primary antibodies were incubated over night at 4 °C and secondary antibodies were incubated 1 h at RT. Sections or coverslips were digitized on an Axioskop epifluorescent microscope (Zeiss) or confocal FV1000 microscope (Olympus). Co-localization analysis of lineage-makers with plexinC1 immunohistochemistry was performed using the image-processing program Fiji^68^. Three to ten random locations along the SGZ were imaged with a confocal FV1000 microscope from at least three hippocampal sections per animal. Images were analysed twice by two observers, once based on marker-positive cells and once based on plexinC1-positive cells as reference cells. Observers were presented with single-channel fluorescent z-stacks for selection of the reference cells. After selection of the reference cells, overlap in immunostaining was assessed based on merged multiple-channel fluorescent z-stacks. Branch number and neurite length were determined with Fiji using the Simple Neurite Tracer plugin. To quantify expression of Sema7A in β1-integrin WT and KO mice the intensity of Sema7A expression was measured in the molecular layer of the DG as described previously^69^. In short, the molecular layer was defined as an ROI. In the ROI, median intensity levels were measured using FIJI. These levels were subtracted from the maximum intensity for an 8-bit image (250) to generate the reciprocal intensity, which was used to determine differences between genotypes.

Primary antibodies used were: sheep anti-plexinC1 (1:40, AF5375 R&D), goat anti-Sema7A (1:100, AF1835 R&D), rat anti-active β1-integrin (1:100, 553715 BD Biosciences), mouse anti-NeuN (1:100, MAB377 Millipore), rat anti-BrdU (1:500, OBT0030 Accurate), rabbit anti-Ki-67 (1:100, ab15580 Abcam), mouse anti-GFAP (1:200, ab10062 Abcam), rabbit anti-GFAP (1:500, AB7260 Sigma), mouse anti-Sox2 (1:200, SC1002 Calbiochem), rabbit anti-TBR2 (1:300, AB2283 Millipore), guinea-pig anti-DCX (1:200, AB2253 Chemicon), rabbit anti-cleaved caspase 3 (1:500, 559565 BD Biosciences), rabbit anti-GFP (1:2,000, ab290 Abcam), chicken anti-GFP (1:400, ab13970 Abcam), rabbit anti-RFP (1:500, 600-401-379 Rockland), rabbit anti-Map2 (1:500, AB5622 Milipore) and mouse anti-Tuj1 (1:500, MMS-435P Covance).

### Non-radioactive *in situ* hybridization

Non-radioactive *in situ* hybridization was performed on coronal brain sections (16 μm) of adult mice using digoxigenin-labelled RNA probes targeting *Sema7A* mRNA (rat *Sema7A*; NC_005107.4, nt 205–941 of coding region) or *β1-integrin* mRNA (mouse β1-integrin; NM_010578, nt 603–1205 of codion region). Probe design and synthesis, and non-radioactive *in situ* hybridization were performed as described previously^3^. In brief, sections were hybridized with 400 ng ml^−1^ RNA in 150 μl hybridization mixture containing 50% deionized formamide, 5 × standard saline citrate (SSC), 1 × Denhardt's solution, 0.25 mg ml^−1^ sonificated salmon sperm DNA, and 0.5 mg ml^−1^ tRNA baker's yeast. After overnight hybridization at 55 °C, slides were washed, incubated with anti-digoxigenin antibodies (1:2,500, 1093274 Boehringer Mannheim) and developed with BCIP/NBT (Boehringer Mannheim). Subsequently, sections were dehydrated and embedded in Entallan (Merck). For co-labelling of BrdU and *Sema7A*, 800 ng ml^−1^ RNA probe was used and Triton X-100 was omitted during antibody incubation steps. Sections were embedded in 95% glycerol to avoid loss of *in situ* hybridization signal. Representative sections were digitized using an Axioskop2 light-microscope (Zeiss).

### Western blotting

Specificity of different anti-plexinC1 and anti-Sema7A antibodies (Supplementary Fig. 1) was examined using western blotting as described^70^. In brief, fresh frozen tissue from *Sema7A*^*−/−*^, *plexinC1*^*−/−*^ or wild-type brains was homogenized in ice-cold RIPA buffer containing 10 mM Tris-buffer pH 7.5, 150 mM NaCl, 0.1% SDS, 1% Triton X-100, 1% Deoxycholate, 0.5 mM EDTA, 1 mM PMSF and complete protease inhibitor (Roche). Protein containing-supernatant was isolated after centrifugation at 12,000 r.p.m. at 4 °C for 15 min. Tissue lysates (20 μg of total protein) were separated by SDS-PAGE and transferred to nitrocellulose membrane. Blocking of the membrane and incubation of antibodies was performed in 5% ELK in TBS-T. To detect protein phosphorylation (Supplementary Fig. 3d–g), neurospheres from WT mice were cultured and treated with Fc (2 nM, R&D) or Sema7A-Fc (2 nM, R&D) proteins for 30 min. Proteins were harvested by homogenization in ice-cold RIPA buffer followed by centrifugation at 12,000 r.p.m. at 4 °C for 15 min. Protein lysates were incubated with sample buffer and 10% β-mercaptoethanol and boiled for 5 min at 95 °C. Samples (30 μg of total protein) were separated in precast 3%-8% SDS-polyacrylamide Tris-acetate gels (Invitrogen). Proteins were transferred to Nitrocellulose membranes (0.2 mm pore-size membranes, Invitrogen). For all membranes, membrane blocking and antibody incubation was performed in 5% ELK in TBS-T. Western blotting was performed using the following antibodies: goat anti-Sema7A (1:1,000, AF1835 R&D), rabbit anti-Sema7A (1:500, ab23578 Abcam), goat anti-Sema7A (1:200, sc67969 Santa Cruz), rabbit anti-plexinC1 (1:200, sc25643 Santa Cruz), sheep anti-plexinC1 (1:200, AF5375 R&D), mouse anti-beta3-tubulin (1:1,000, MMS-435 P Convance), mouse anti-P-cofilin (1:500, sc271923 Santa Cruz Biotechnology), rabbit anti-cofilin (1:500, sc33779 Santa Cruz Biotechnology), rabbit anti-integrin β1 (1:500, sc8978 Santa Cruz Biotechnology), rabbit anti-P-integrin β1 (1:500, ab5189 Abcam) followed by incubation with HRP-conjugated secondary antibody (anti-rabbit (1:10,000), anti-goat (1:15,000) or anti-mouse (1:30,000)). After incubation with Pierce SuperSignal chemiluminescence kit, blots were exposed to ECL film (Pierce), developed and digitized using an Epson flatbed scanner (Perfection 4990, Epson America) for tissue lysates or detected via enhanced chemiluminescence (NEL101, PerkinElmer) for neurosphere lysates.

### Isolation and culturing of aNPCs from the adult DG

Isolation and culture of adult NPCs (aNPCs) was performed as described previously^26^,^28^. aNPCs were isolated from 8–10-week-old *plexinC1*^*−/−*^, *Sema7A*^*−/−*^ or wild-type mice. The whole hippocampus was rolled out followed by microdissection of the DG. The isolated DG was dissociated by enzymatic digestion using the MACS Neural Tissue Dissociation kit (Miltenyi Biotech). Dissociated cells were passed through a 40 μm cell-strainer and seeded as a single-cell suspension in medium containing glutaMAX DMEM/F-12 (Gibco), 20 ng ml^−1^ EGF (Invitrogen), 20 ng ml^−1^ FGF (Gibco), B-27 (Gibco) and penicillin/streptomycin (Gibco). Cultures were kept in a 5% CO_2_ incubator at 37 °C. Neurospheres formed by proliferating aNPCs were passaged at a diameter of ±150–200 μm. Neurospheres were dissociated in Trypsin LE Express (Gibco) for 5 min at 37 °C, passed through a 40 μm filter and cultured as a single-cell suspension. All differentiation and proliferation assays were performed using aNPCs with a passage number of 0–10 to avoid undesired side effects due to prolonged *in vitro* culture.

### RT-PCR

Passage 10 neurospheres were collected and mRNA was isolated using the RNeasy micro kit (Qiagen). One-step RT-PCR was conducted, as described previously^3^ on 100 ng μl^−1^ RNA using the following primers: *Sema7A* 5′-CGTGTATTCGCTTGGTGACAT-3′, 5′-GTGGGTATGGGCTGCTTTTT-3′ (product size of 120 bp) (ref. 71), *β1-integrin* 5′-AATTTGCAACTGGTTTCCTG-3′, 5′-GTTTGAGAGCCTCTGGGATT-3′ (product size of 260 bp), *α1-integrin* 5′-CATCTGGCTTCTCACCGTTA-3′, 5′-CTCTCTCTCCCAACTGGACAC-3′ (product size of 219 bp), *αv-integrin* 5′-GTCCTCCAGGATGTTTCTCC-3′, 5′-ACCTCACAGAGGCTCCAAAC-3′ (product size of 209 bp), and *plexinC1* 5′-TCTGGAACGGCAGTGTCTAC-3′, 5′-GCCTGGATCTCACTCATCCT-3′ (product size of 298 bp). *HPRT* primers were from Quantitect Primer Assay (product size of 107 bp, Qiagen). PCR cycle-sequence was as follows: 5 min/95 °C, 30 s/95 °C, 30 s/54 °C, 45 s/72 °C (for 30, 35 and 40 cycles), 7 min/72 °C.

### Purification of AP-Fc-tagged Sema7A

Recombinant AP-Sema7A-Fc and AP-Fc control proteins^5^ were freshly purified for each experiment as described previously^9^. HEK293 cells were cultured in 175 cm^2^ flasks at 70–80% confluency and transiently transfected with 32 μg DNA vector per flask (Sema7A-AP-Fc or AP-Fc) using PEI (Polyscience). Transfected cells were cultured in OptiMEM medium for 36–48 h before harvesting. Then medium was filtered and concentrated using filter spin column (50 kDa, Millipore) and Ultrafree 0.5 ml centrifugal filters (10 kDa, Millipore), respectively. Recombinant protein concentration was determined by measuring alkaline-phosphatase activity.

### *In vitro* proliferation and differentiation assays

For proliferation assays on aNPCs, passage 4–10 neurospheres were dissociated, seeded as a single-cell suspension at a density of 5,000 cells well^−1^ (24-wells plate, 500 μl growth volume) and allowed to form neurospheres for 8–10 days. Medium containing recombinant Sema7A-AP-Fc (2 nM) or AP-Fc (2 nM) was refreshed every other day. Experiments were terminated by addition of an equal volume, relative to medium, of 8% PFA in PBS and stored for analysis at 4 °C. Neurosphere number and diameter were determined using an upright light microscope (AxioVert, Zeiss) and the image-processing program Fiji.

For differentiation assays, small neurospheres (diameter of ∼50 μm) were seeded onto acid-etched coverslips coated with poly-L-lysine (500 μg ml^−1^, Sigma-Aldrich) and laminin (40 μg ml^−1^, Sigma-Aldrich), and cultured in aNPC medium for 36 h. Subsequently, medium was changed to differentiation medium consisting of Neurobasal medium (Gibco) supplemented with 200 mM L-Glut, B27, 1.8 mM Hepes, and penicillin/streptomycin. Differentiation medium was supplemented with recombinant Sema7A-Fc (2 nM, R&D) or Fc (2 nM, R&D) and anti-rat IgG-control (25 μg ml^−1^, 553968 BD bioscience) or anti-rat CD29 (25 μg ml^−1^, 555002 BD biosciences) every other day. After 10 days, coverslips were processed for immunohistochemistry.

### Stereology

BrdU-positive cells were counted as described previously^27^, using a brightfield microscope (Zeiss) and Neurolucida (MBF Bioscience) and Fiji software. Cells were counted throughout the anterior-posterior extent of the GC layer in a series of 40 μm coronal sections spaced 240 μm apart. Total cell number in the DG per hippocampus was calculated based on the multiply of counted BrdU-positive cells, corrected for DG volume estimate and sampling frequency. Systematic random sampling with a dissector (625 μm^2^) was used to determine the cell density of the DG granule layer (Supplementary Fig. 6e). GCs were counted in a series of 40 μm coronal sections with a vibratome advance of 240 μm. Total cell density was determined by correcting the total sum of counted GCs by dissector frame, sum of dissectors used, mean thickness of slice, vibratome advance and number of points associated with the dissector frame.

### Morphometric analysis of adult-born GCs in the adult DG

Stained sections from MMLV-injected brains were scanned on a confocal microscope (FV1000, Olympus) using × 40 (UPlanSApo UIS2 0.95 NA, Olympus) and oil-immersion × 100 (UPlanSApo UIS2 1.40 NA, Olympus) objectives. Z-stack images were captured at a resolution of: × 40 objective x/y-plane: 3.23 pixels μm^−1^, z-plane: 1 μm and × 100 objective x/y-plane: 8.06 pixels μm^−1^, z-plane: 0.40 μm. Analysed nGCs extended their dendrites within the specimen section, avoiding the outer 5 μm of the section-depth. Images captured of GFP/RFP-positive adult-born GCs by using the × 40 objective were used to identify (1) the inner molecular layer (IML), which was defined as the first 50 μm from and parallel to the outer border of DAPI-positive cells, and (2) the outer molecular layer (OML), which was defined as the last 70 μm from and parallel to the outer border of the molecular layer. GFP-positive dendrites within the IML and OML were imaged, by using a × 100 objective for spine density analysis. Analysis of dendritic branching was performed by using Neurolucida (MBF Bioscience) and spine density analysis and migration distance was performed using Fiji. Mushroom-like spines were defined as described earlier^40^,^41^, that is, spines with a head diameter much greater than the diameter of the neck were judged mushroom-like.

### Electrophysiology

Mice (WT and *Sema7A*^*−/−*^) were injected at P54-P58 with 1 μl of MMLV virus encoding CAG-GFP in DG (as described in the section ‘Stereotaxic injections'). Coronal brain slices were prepared 56–60 days later from mice sedated with isoflurane and perfused transcardially with ice-cold ACSF containing (in mM): NaCl (127), NaHCO_3_ (25), D-glucose (25), KCl (2.5), MgCl_2_ (1), CaCl_2_ (2), and NaH_2_PO_4_ (1.25), pH 7.25–7.35, ∼310 mOsm, and bubbled with 95% O_2_/ 5% CO_2_, as described previously^37^,^72^. The brain was removed and placed into ice-cold cutting solution containing (in mM): choline chloride (110), NaHCO_3_ (25), D-glucose (25), sodium ascorbate (11.5), MgCl_2_ (7), sodium pyruvate (3), KCl (2.5), NaH_2_PO_4_ (1.25) and CaCl_2_ (0.5). The ventral side of the brain was placed onto a 10° aluminium wedge, anterior side facing the inclining part of the slope and 300 μm thick horizontal slices were vibratome sectioned (Leica 1200S). Slices were incubated in oxygenated ACSF for 30 min at 34 °C, and then maintained in an oxygenated holding chamber for at least 30 min at RT before commencing recording.

For recording of intrinsic properties of GCs oxygenated ACSF was supplemented with (in μM) GABA_B_-receptor antagonist CGP 52432 (10), GABA_A_-receptor antagonist SR 95531 (10), AMPA receptor antagonist NBQX (10) and NMDA receptor antagonist CPP (5). For recording of mEPSCs oxygenated ACSF was supplemented with (in μM) Na^+^ channel blocker TTX (1) and GABA_B_-receptor antagonist CGP 52432 (10), GABA_A_-receptor antagonist SR 95531 (10), and NMDA receptor antagonist CPP (5). All compounds were purchased from Tocris.

Borosilicate pipettes (2.8–6 MΩ; Warner Instruments) were filled with potassium gluconate-based internal solution ((in mM) potassium sulfonate (120), KCl (20), MgCl_2_ (4), NaCl (5), Hepes (10), EGTA (0.1), Na_2_-phosphocreatine (10), Na_2_ATP (4), Na_2_GTP (0.3); pH 7.35–7.40; 290 mOsm) for recording of intrinsic properties and cesium methanesulfonate-based internal solution ((in mM) cesium methanesulfonate (135), NaCl (8), Hepes (10), EGTA (0.5), Na_2_ATP (4), Na_2_GTP (0.3); pH 7.35–7.40; 290 mOsm) for mEPSC recording. Both internal solutions contained 3 mg ml^−1^ biocytin (Sigma) and 10 μM Alexa Fluor 594 dye (Invitrogen).

Whole-cell patch-clamp recordings were acquired at 10 kHz using a Multiclamp 700B with an online 2 kHz low pass filter (Molecular Devices) and *Ephus* software (www.ephus.org). Data analysis was performed in Matlab, R (http://cran.r-project.org), Igor Pro (Wavemetrics) and Excel (Microsoft). Recordings were corrected for a liquid junction potential of 7.6 and 15 mV, for the potassium gluconate-based and the cesium methanesulfonate-based internal solution, respectively. Series resistance was typically 15–35 MOhm. Resting membrane potentials were measured immediately after break-in. *R*_input_ was measured using a linear-regression of voltage deflections (±15 mV from resting potential) in response to 200 ms current steps of eight different amplitudes (increments, 25 pA). The time constant (Tau_m_) was calculated fitting a single exponential function to the mean voltage responses to three hyperpolarizing and three depolarizing current steps (−25 pA and 25 pA, 200 ms). Membrane capacitance (*C*_m_) was calculated according to *C*_m_=*Tau*_m_/*R*_m_. mEPSCs were detected using template fitting, generated by manual setting of rise- and decay-time constants, in a custom-written Matlab routine (by Haining Zhong). Visual validation was performed after automatic detection of mEPSCs. Rise- and decay time were calculated based on 10-90% of mEPSC peak value. Instantaneous frequency of individual events was calculated based on the inverse of the time interval (in seconds) to the previous event. Amplitude and instantaneous frequency histograms of mEPSC were fitted with a single lognormal distribution using the following equation:


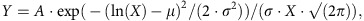


where *Y* represents number of instantaneous frequency/amplitude events, *A* relative area under the curve, *X* the measured instantaneous frequency/amplitude, *μ* is the mean and *σ* is the s.d. of the underlying normal distribution. The mean of the lognormal distribution (*m*) was then calculated using the equation:


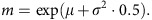


Action potential (AP) kinetics was determined on isolated APs elicited at rheobase current injection. AP threshold was determined during a current ramp (165 pA s^−1^), for which AP onset was defined as *V*_membrane_ s^−1^>1 V s^−1^. Spike analysis was performed automatically by selecting individual APs based on AP onset (*V*_membrane_ s^−1^>1 V s^−1^), and peak amplitude (*V*_membrane_>0 mV), and subsequent manual validation. Inter-spike-interval was calculated for each AP and used for firing rate and firing adaptation index, 1−(*F*_last_/*F*_initial_) calculation, for which *F*_initial_ is the averaged inter-spike-interval of the first two spike intervals and *F*_last_ is the averaged inter-spike-interval of the last three spike intervals.

### Blinding methods and statistical analyses

To avoid observer bias, all quantitative assessments in this manuscript were performed while being unaware of genotype or condition. During analysis, raw data were named in a descriptive way, without revealing experimental group information. (Fluorescent) microscopic imaging was always done with the same settings within experiments, and analysis techniques were standardized.

A single person analysed all data obtained within the experiments. All experiments were replicated at least three times. All statistical analyses were performed using Excel (Microsoft Office, Microsoft, USA), Igor Pro (Version 6.0, Wavemetrics, USA), SPSS (Version 21.0, IBM Inc., USA), or R (version 3.1.2, http://cran.r-project.org). Thresholds of significance were set at *α*=0.05. Sholl-analyses were statistically tested using repeated-measures ANOVA, with Student's *t*-test *post-hoc* per radii. Two-way ANOVA with *post-hoc* Student's *t*-test was used where multi-factorial comparisons were made and specifically mentioned in the text. Homogeneity of variance was tested with the Levene's test. Paired-testing was performed where littermate-controls or technical-replicate were compared and specifically mentioned in the text, otherwise unpaired Student's *t*-test was used. The number of experiments performed with independent cultures or mice, (*n*) unless otherwise stated, is indicated in the legends. Error bars represent s.e.m.

### Data availability

The data sets generated and analysed during the current study are available from the corresponding author on request.

## Additional information

**How to cite this article:** Jongbloets, B. C. *et al*. Stage-specific functions of Semaphorin7A during adult hippocampal neurogenesis rely on distinct receptors. *Nat. Commun.* **8,** 14666 doi: 10.1038/ncomms14666 (2017).

**Publisher's note:** Springer Nature remains neutral with regard to jurisdictional claims in published maps and institutional affiliations.

## Supplementary Material

Supplementary Information Supplementary Figures 1-10 and Supplementary Tables 1-2
