# Supplementary material for: Stage-specific functions of Semaphorin7A during adult hippocampal neurogenesis rely on distinct receptors
Source: Nat Commun. 2017 Mar 10;8:14666. doi: 10.1038/ncomms14666 (PMC5353663; doi:10.1038/ncomms14666)
Supplement: Supplementary Information — Supplementary Figures 1-10 and Supplementary Tables 1-2 [file ncomms14666-s1.pdf]

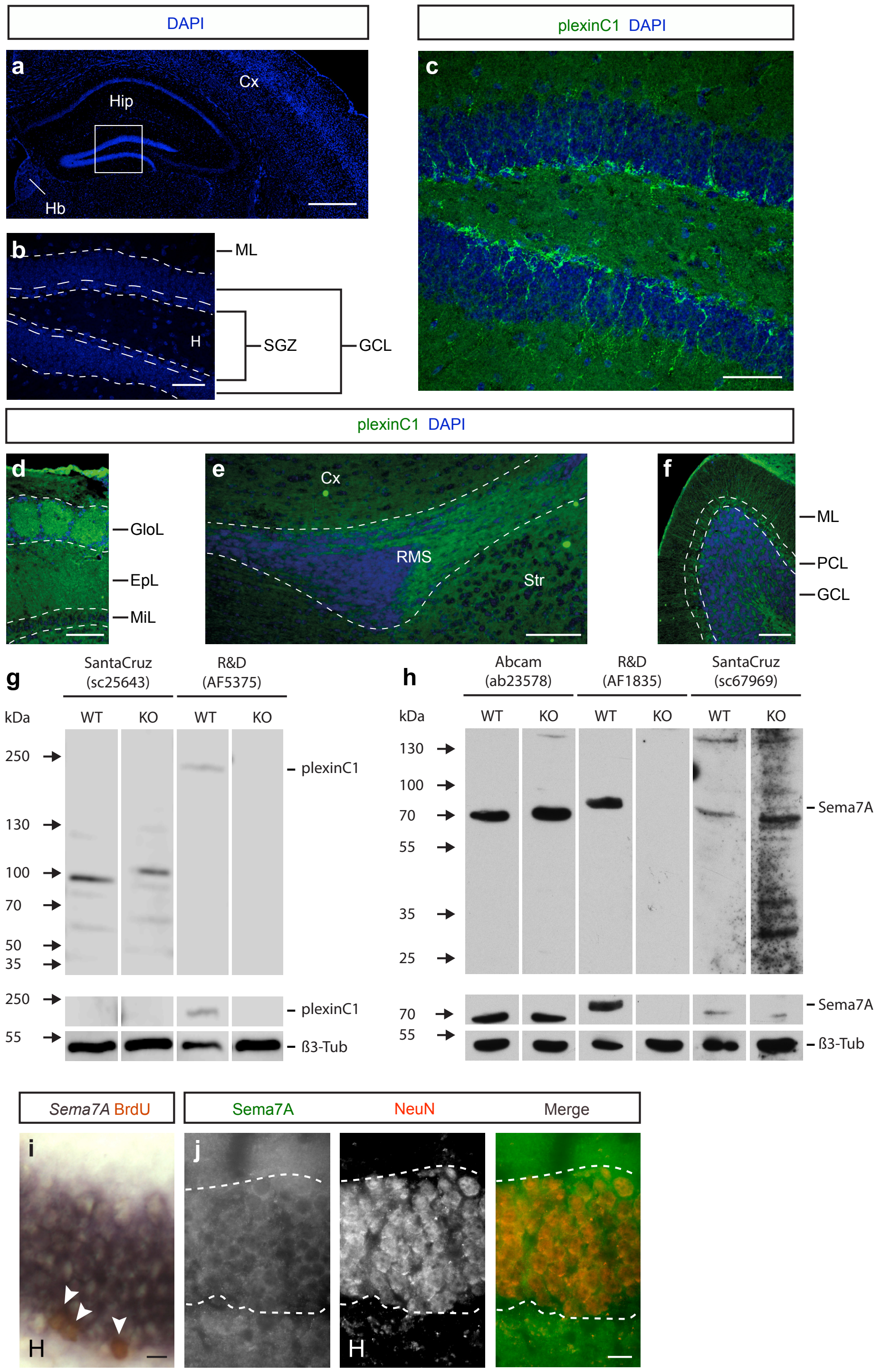

Supplementary Figure 1. Analysis of antibody specificity and plexinC1 - Sema7A expression in the adult dentate gyrus.

(**a, b**) Mature granule cells reside within the two blades of the granular cell layer (GCL) in the dentate gyrus (DG) of the hippocampus (Hip). A neurogenic niche exists beneath the GCL, called the subgranular zone (SGZ). Boxed area in **a** is shown at higher magnification in **b**. DAPI in blue. Cx, cortex; H, hilus; Hb, habenula; ML, molecular layer.

(**c-f**) Immunohistochemistry for plexinC1 on coronal (**c**) and sagittal (**d-f**) sections of the adult mouse brain. In the adult DG, shown in panel c, plexinC1 expression is restricted to a subpopulation of cells beneath the GCL.(**d**) Expression of plexinC1 is largely confined to the glomerular layer (GloL) of the olfactory bulbs, while the external plexiform layer (EpL) and the mitral cell layer (MiL) show sparse labelling. (**e**) Progenitors from the subventricular zone migrate to the olfactory bulbs through the rostral migratory stream (RMS), situated between the Cx and the striatum (Str). Robust expression of plexinC1 is observed in the RMS. (**f**) Purkinje cells and granule cells situated in the cerebellar purkinje cell layer (PCL) and GCL, respectively, express high levels of plexinC1 protein. DAPI in blue.

(**g**) Western blotting of *plexinC1*$^{-/-}$ (KO) and *plexinC1*$^{+/+}$ (WT) brain lysates with different anti-plexinC1 antibodies. Upper panels show whole immunoblots, lower panels show cropped immunoblots at predicted size (± 206 kDa) and tubulin loading-control (ß3-Tub, ± 50 kDa). The R&D antibody specifically detects plexinC1.

(**h**) Western blotting of *Sema7A*$^{-/-}$ (KO) and *Sema7A*$^{+/+}$ brain lysates with different anti-Sema7A antibodies. Upper panels show whole immunoblots, lower panels show cropped immunoblots at predicted size (±80 kDa) and tubulin loading-control (ß3-Tub, ± 50kDa). The R&D antibody specifically detects Sema7A.

(**i**) *In situ* hybridization for *Sema7A* (purple) combined with immunohistochemistry for BrdU (brown) in the adult DG at 2 hrs after BrdU injection. Proliferating, BrdU-positive cells (arrowheads) express *Sema7A*.

(**j**) Double immunohistochemistry for Sema7A and NeuN, a marker for mature granule cells, on sections of the adult DG. Sema7A is expressed throughout the DG GCL (indicated by dotted lines) in NeuN-positive and -negative cells. Scale bars: **a** 250 μm; **b, c** 50 μm; **e**-**h** 100 μm; **i, j** 10 μm.

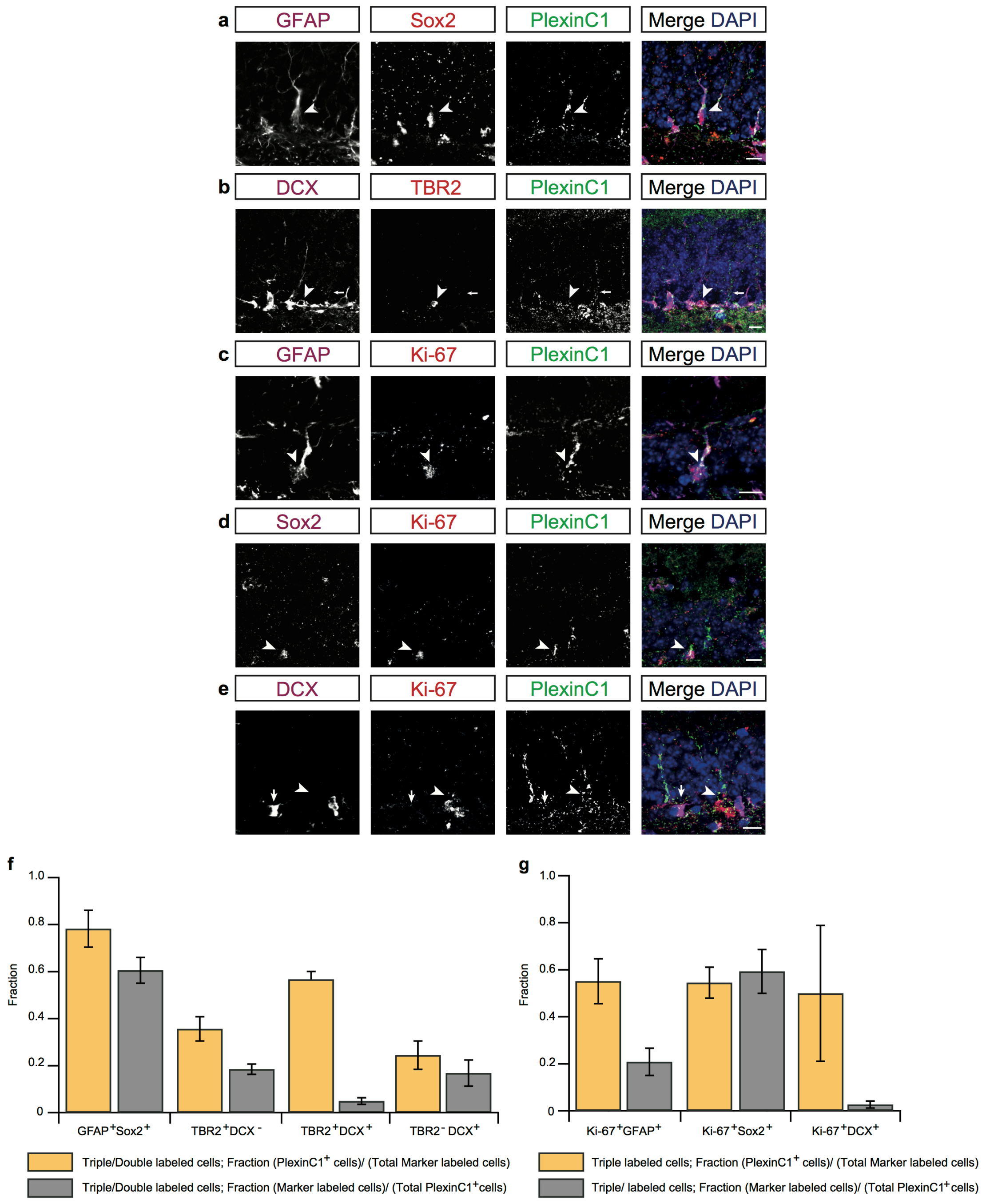

Supplementary Figure 2. PlexinC1 is enriched in radial glial cells and early immediate progenitor cells of the dentate gyrus subgranular zone.

(**a-e**) Triple immunohistochemistry for plexinC1 and different marker proteins.

(**f**, **g**) Quantification of the fraction of cells expressing specific markers that also express plexinC1 (yellow) or the fraction of plexinC1-positive cells that also express the indicated marker proteins (grey). A large fraction of cells expressing glial fibrillary acidic protein (GFAP) and sex determining region Y-box 2 (Sox2) also express plexinC1. Similarly, a large portion of plexinC1-positive cells express GFAP and Sox2. A small fraction of immediate progenitors (t-box brain 2 (TBR2)-positive and doublecortin (DCX)-negative), neuroblasts (TBR2-positive and DCX-positive), and immature neurons (TBR2-negative and DCX-positive) express plexinC1. A small fraction of plexinC1-positive cells are identified as immediate progenitors, neuroblasts or immature neurons. (**g**) A large fraction cells positive for Ki-67 and GFAP, Sox2 or DCX express plexinC1. A large number of plexinC1-positive cells show Ki-67 and Sox2 co-expression. In contrast, small numbers of plexinC1-positive cells also express Ki-67 and GFAP or Ki-67 and DCX. All data are obtained from n = 3 mice and presented as means ± SEM. Scale bar: 10 µm.

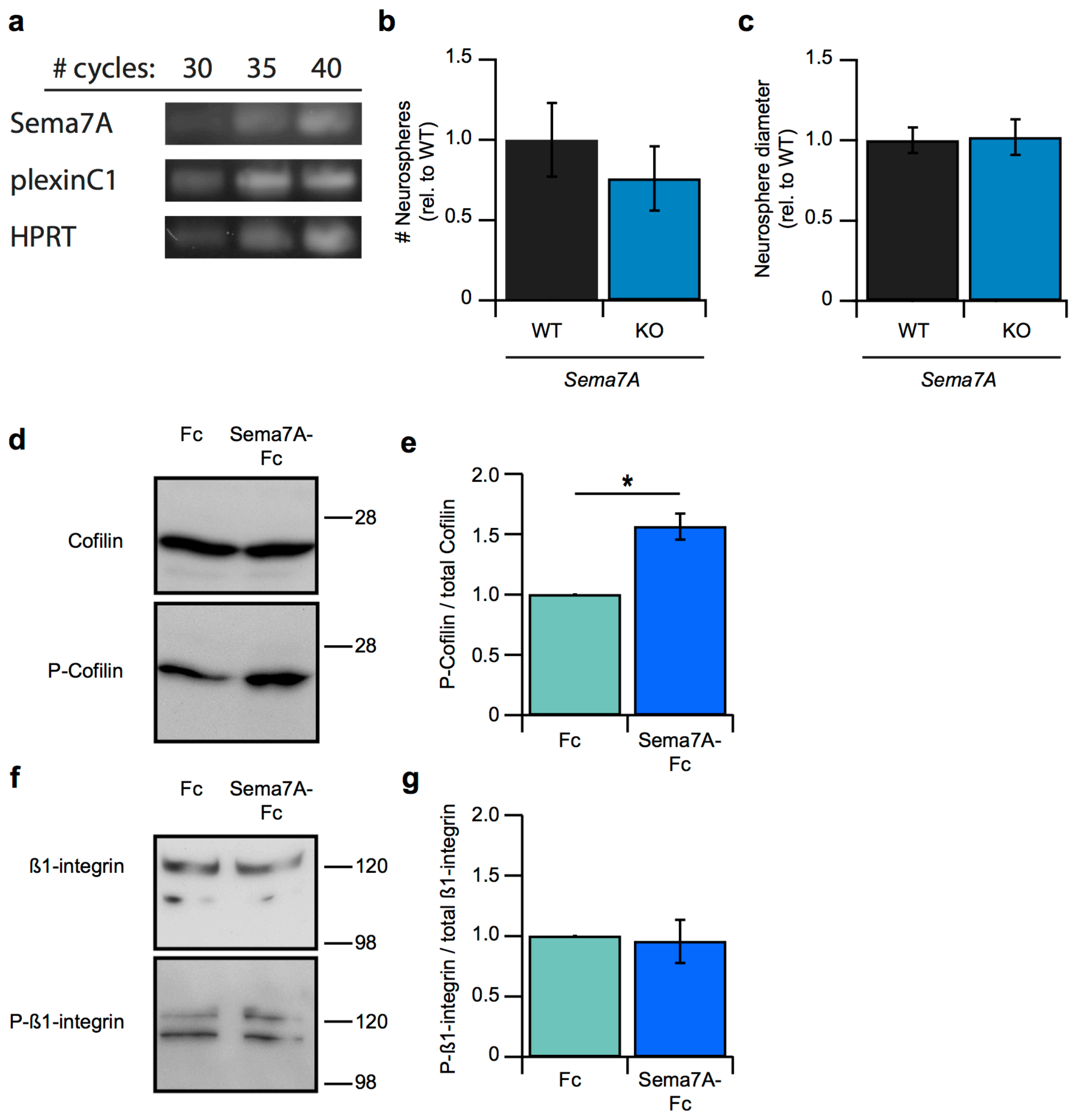

Supplementary Figure 3. Adult subgranular zone-derived neurospheres do not cell-autonomously require Sema7A for growth and Sema7A induces phosphorylation of cofilin but not β1-integrins in neurospheres.

(**a**) RT-PCR for *Sema7A*, *plexinC1*, and the house-keeping gene *HPRT* in passage 10 neurospheres.

(**b**, **c**) Neurosphere number and size are unchanged in *Sema7A*$^{-/-}$ (KO) mouse cultures (Littermate-paired Student's *t*-test: $n = 5$ mice, $P_{(number)} = 0.4515$, $P_{(diameter)} = 0.8900$). WT, *Sema7A*$^{+/+}$.

(**d-g**) Wild-type neurospheres were treated with exogenous Sema7A-Fc (2 nM) or Fc (2 nM) for 30 minutes followed by cell lysis and western blot analysis using the indicated antibodies (**d, f**). (**e**, **f**) Quantification of band intensity of experiments as shown in **d** and **f** was performed by scanning densitometry. Signals were normalized to an internal loading control and then to the Fc condition and are represented as the fraction of phosphorylated protein of total protein ($n = 3$ independent neurosphere cultures, replicate-paired Student's *t*-test: $P_{(P\text{-}\beta 1\text{-}integrin)} = 0.827$). All data are presented as means ± SEM, * $P < 0.05$. Sema7A treatment leads to an increase in the phosphorylation of cofilin but not β1-integrins.

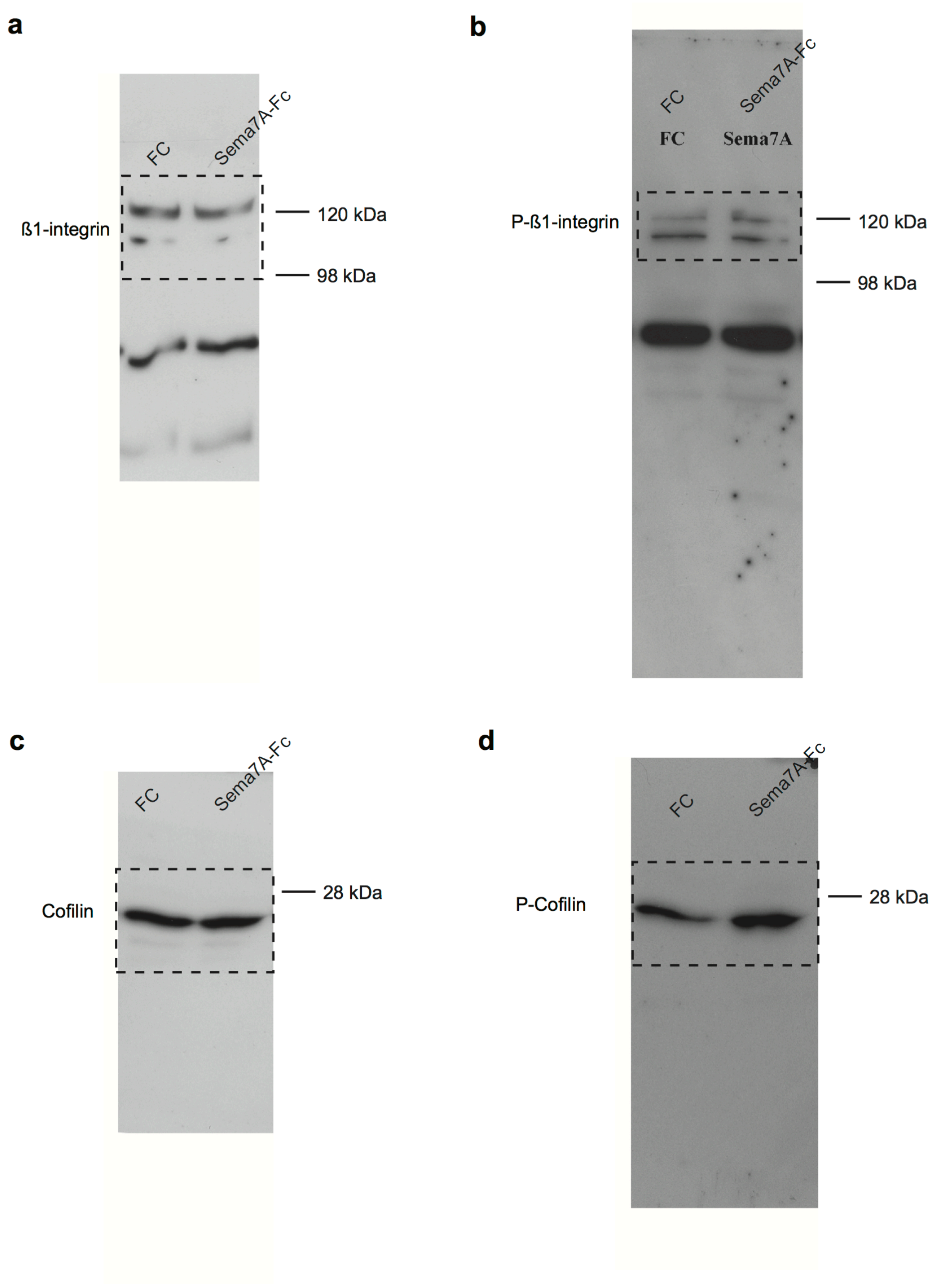

Supplementary Figure 4. Uncropped western blots from the experiments shown in Supplementary Fig. 3.

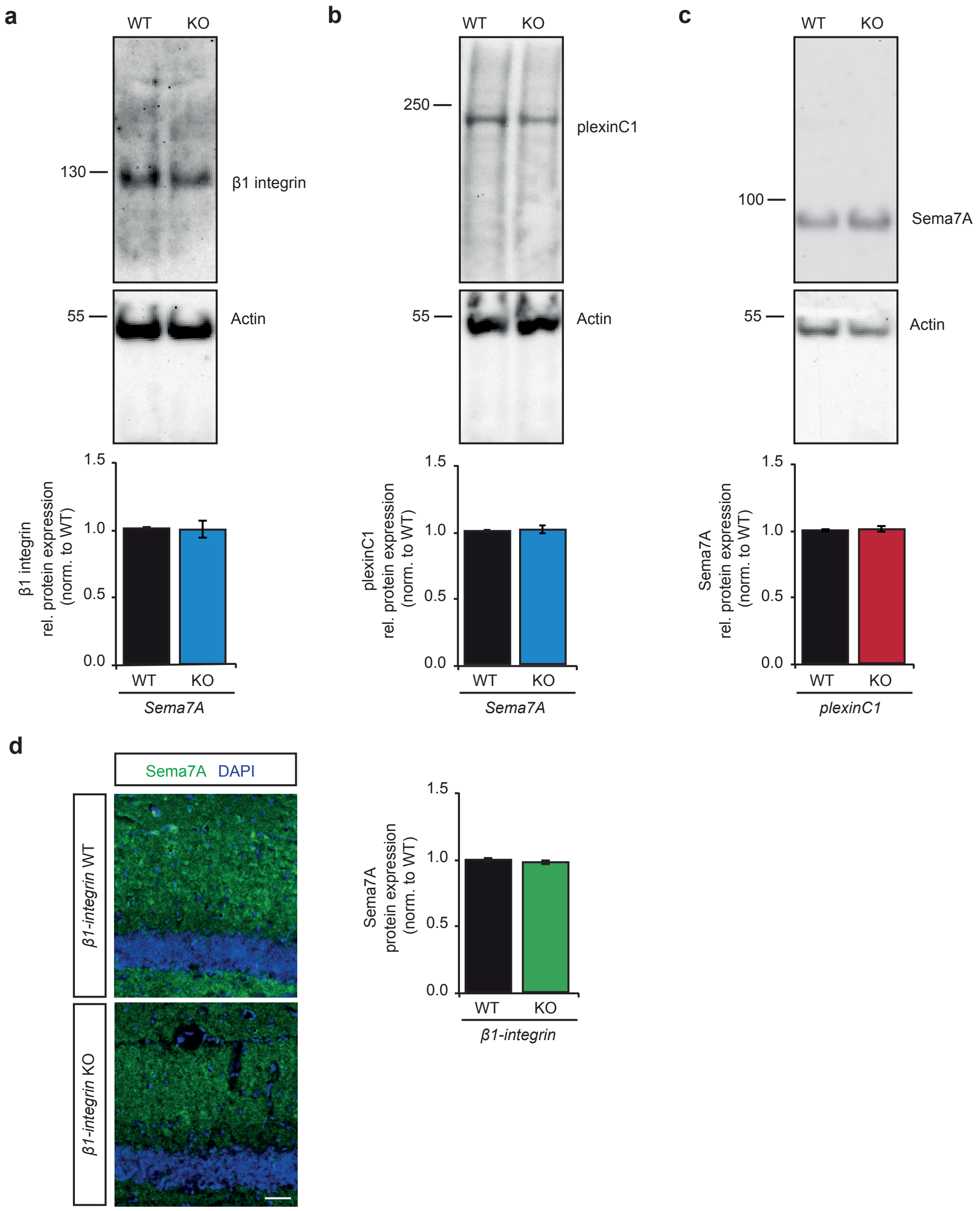

Supplementary Figure 5. Expression analysis of Sema7A, plexinC1 and ß1-integrin in different knockout mice.

Expression of plexinC1 (a) and β1-integrin (b) was determined in adult Sema7A$^{+/+}$ (WT) and Sema7A$^{-/-}$ (KO) brain lysates using Western blotting. Upper panels show bands for β1-integrin and plexinC1 at the predicted size. Lower panels show actin as a

loading control. Graphs show quantification of normalized levels between genotypes. No differences were observed (Student's *t*-test: $P_{(\text{plexinC1})} = 0.5936$ and $P_{(\beta\text{1-integrin})} = 0.7698$). Data represent $n = 3$ (mice) and are presented as means ± SD.

(c) Brain lysates of plexinC1 $^{+/+}$ (WT) and plexinC1$^{-/-}$ (KO) were used to determine Sema7A expression by Western blotting. Upper panel shows a band for Sema7A at the predicted size. Lower panel shows actin as a loading control. Graph shows quantification of normalized Sema7A levels between genotypes. No difference was detected. (Student's *t*-test: $P_{(\text{Sema7A in plexinC1})} = 0.5499$). Data represent n = 3 (mice) and are presented as means ± SEM.

(d) Sections of nestin-Cre;β1-integrin$^{+/+}$ (WT) and nestin-Cre;β1-integrin$^{fl/fl}$ (KO) mice were immunostained for Sema7A and DAPI (left panel). Images show dentate gyrus (strongly labeled DAPI-positive cell bodies in blue in the lower part of the image and molecular layer on top of the GC layer in upper part of the image (strongly stained for Sema7A in green). Right panel shows quantification of the intensity of normalized Sema7A immunostaining. No difference was found between WT and KO immunostainings (Student's *t*-test $P_{(\text{Sema7A in }\beta\text{1-integrin})} = 0.2540$). Data are obtained from $n = 3$ mice and presented as means ± SEM. Scale bar: 50 µm.

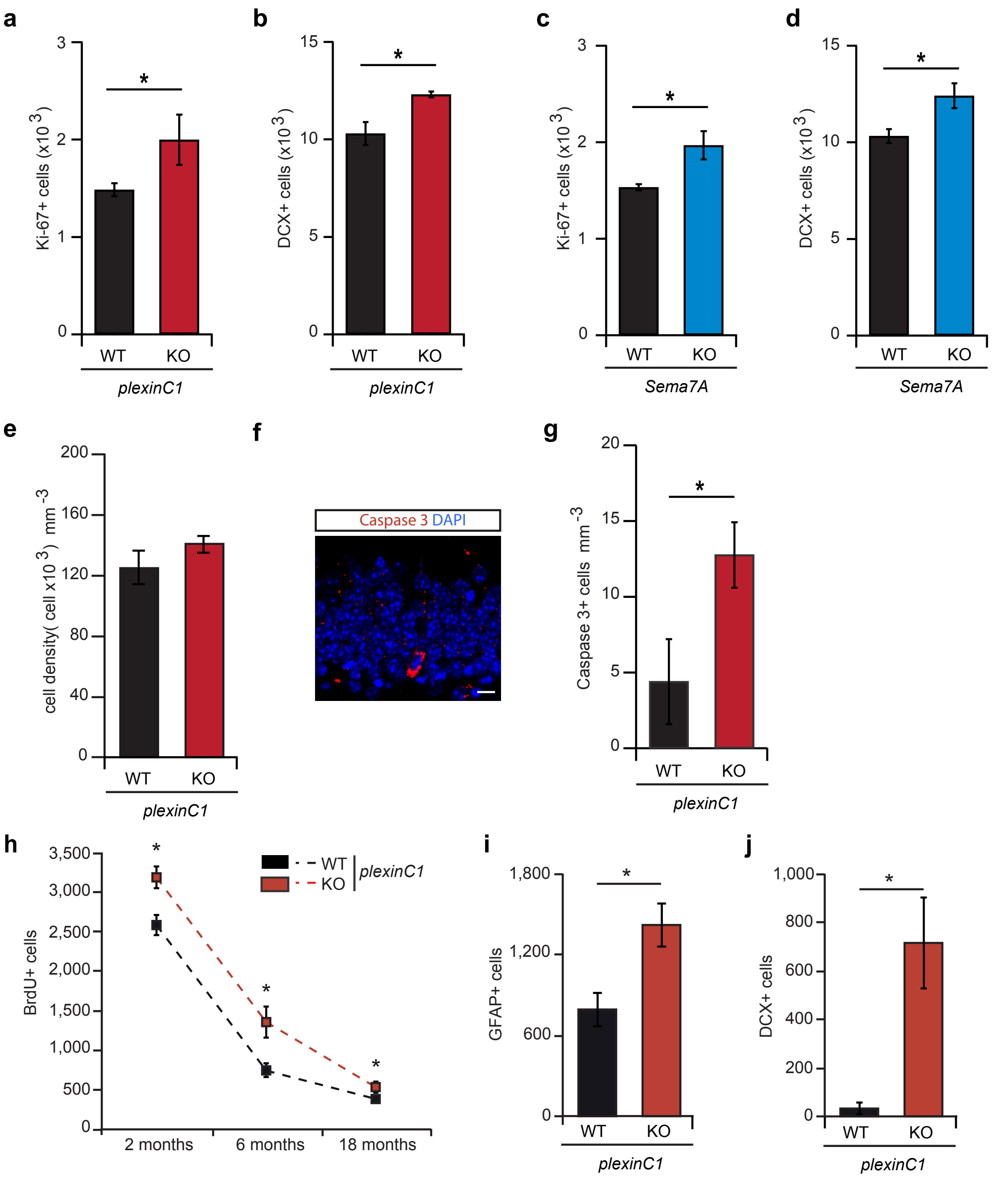

Supplementary Figure 6. Increased cell proliferation in the subgranular zone of the DG in *plexinC1* and *Sema7A*-deficient mice.

(**a-d**) Quantification of the number of Ki-67$^+$ or DCX$^+$ cells in *plexinC1*$^{-/-}$ mice (**a**, **b**; KO), *Sema7A*$^{-/-}$ mice (**c**, **d**; KO) or littermate controls (WT)(*n* = 3 *plexinC1*$^{+/+}$, *n* = 4 *plexinC1*$^{-/-}$, *Sema7A*$^{+/+}$, and *Sema7A*$^{-/-}$ mice; Student's *t*-test). The number of Ki-67$^+$ and DCX$^+$ cells is increased in *plexinC1*$^{-/-}$ and *Sema7A*$^{-/-}$ mice.

(**e**) Cell density measurements in the dentate gyrus (DG) granule cell layer (GCL) in *plexinC1*$^{-/-}$ mice or littermate controls (WT) (*n = 3* mice*,* Student's *t*-test, $P = 0.141$). Granule cell density is unchanged in *plexinC1*$^{-/-}$ mice.

(**f**) Immunohistochemistry for cleaved caspase-3 in the DG GCL. DAPI in blue.

(**g**) Quantification of experiments as in **f** shows that cleaved caspase-3 levels are increased in *plexinC1*$^{-/-}$ mice compared to littermate controls (WT; *n* = 6 mice, Student's *t*-test). * $P < 0.05$.

(**h**) Quantification of BrdU-positive cells in *plexinC1*$^{-/-}$ mice and littermate controls (WT) at 2, 6 and 18 months of age. *PlexinC1*$^{-/-}$ mice show increased levels of BrdU at all ages examined (*n = 7 plexinC1*$^{-/-}$ and *n* = 4 *plexinC1*$^{+/+}$ mice for 2 months; *n = 3 plexinC1*$^{+/+}$ and *plexinC1* $^{-/-}$ mice for 6 months; *n = 4 plexinC1*$^{-/-}$ and *n* = 5 *plexinC1*$^{+/+}$ mice for 18 months, two-way ANOVA dpi x genotype $F_{(2, 19)} = 4.325$ $P = 0.028$, *post-hoc t*-tests).

(**i, j**) Quantification of the number of GFAP$^+$ or DCX$^+$ cells in 18 months old *plexinC1*$^{-/-}$ mice shows an increased number of GFAP-positive (**i**) (*n* = 3 mice, Student's *t*-test) and DCX+ (**j**) (*n* = 3 mice, Student's *t*-test) cells in *plexinC1*$^{-/-}$ mice. All data are presented as means ± SEM. Scale bar: 10 µm.

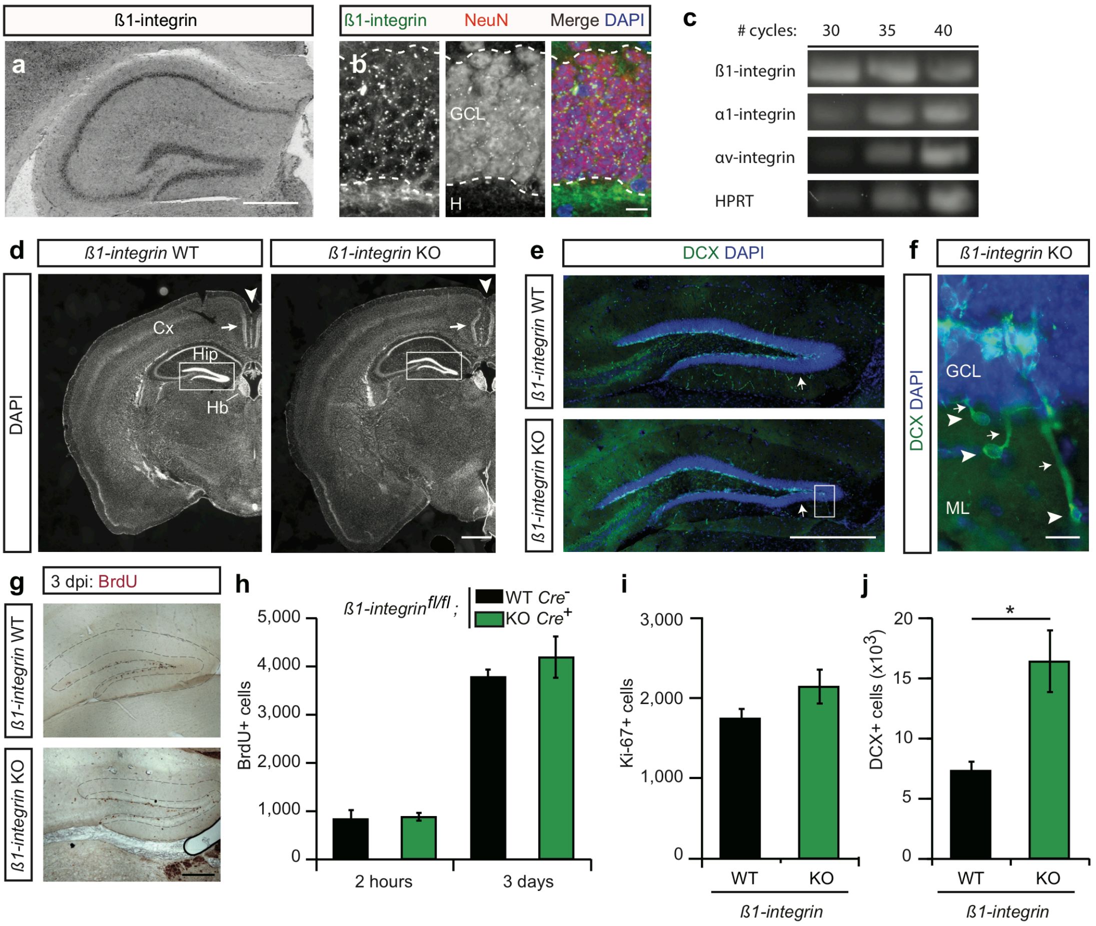

Supplementary Figure 7. Expression of ß1-integrin in the adult DG is required for proper development and the number of immature granule cells.

(**a**) *In situ* hybridization for *ß1-integrin* reveals broad expression in the adult hippocampus.

(**b**) Double immunohistochemistry using an antibody against active ß1-integrin (green) and NeuN (red) shows that the majority of granule cells, both NeuN-positive and -negative, express ß1-integrin protein. DAPI in blue. GCL, granular cell layer; H, hilus

(**c**) RT-PCR for *ß1*-, *α1*-, and *αv-integrin* receptor subunits and the housekeeping gene HPRT, as shown in Supplementary Fig. 3a, from passage 10 neurospheres.

(**d**) DAPI-stained coronal sections from *ß1-integrin*$^{fl/fl}$*;nestin-Cre*$^{+/-}$ (KO) brains show abnormal cell alignment in the cortex (Cx, arrow) and hemisphere separation (arrowhead) as compared to *ß1-integrin*$^{fl/fl}$*;nestin-Cre*$^{-/-}$ control mice (WT). Boxed area in **d** is shown at higher magnification in **e**. Hip, hippocampus; Hb, habenula.

(**e**) Immunostaining for immature granule cells (DCX, green) and cell nuclei (DAPI, blue) in the DG in *ß1-integrin*$^{fl/fl}$*;nestin-Cre*$^{+/-}$ (KO) mice shows disrupted alignment of the GCL within the infrapyramidal blade (arrow) compared to *ß1-integrin*$^{fl/fl}$*;nestin-Cre*$^{-/-}$ (WT).

(**f**) Higher magnification of the boxed area in **e** reveals misplaced DCX^{+} cells (arrowheads) in the molecular layer (ML) of the DG with disoriented dendrites (arrows) facing the GCL.

(**g**) Representative examples of BrdU immunostaining in *ß1-integrin*$^{fl/fl}$*;nestin-Cre*$^{+/-}$ (KO) and *ß1-integrin*$^{fl/fl}$*;nestin-Cre*$^{-/-}$ (WT) mice.

(**h**) Quantification of the number of BrdU-positive cells at 2 hours and 3 days post-injection in the DG of *ß1-integrin*$^{fl/fl}$*;nestin-Cre*$^{+/-}$ (KO) and *ß1-integrin*$^{fl/fl}$*;nestin-Cre*$^{-/-}$ (WT) mice (n = 3 mice, Student's t-test: $P_{(2\ hours)}$ = 0.8302 $P_{(3\ days)}$ = 0.4199).

(**i, j**) Quantification of the number of Ki-67^{+} or DCX^{+} cells in *ß1-integrin*$^{fl/fl}$*;nestin-Cre*$^{+/-}$ (KO) and *ß1-integrin*$^{fl/fl}$*;nestin-Cre*$^{-/-}$ (WT) mice (($n_{(Ki\text{-}67)}$ = 9 WT, $n_{(Ki\text{-}67)}$ = 8 KO, $n_{(DCX)}$ = 3 WT and KO mice: Student's t-test: $P_{(Ki\text{-}67)}$ = 0.1046). The number of DCX-positive cells is increased in KO mice. Data are presented as means ± SEM. * $P < 0.05$. Scale bars: **d**, **e** 500 µm; **f** 100 µm; **g** 250 µm.

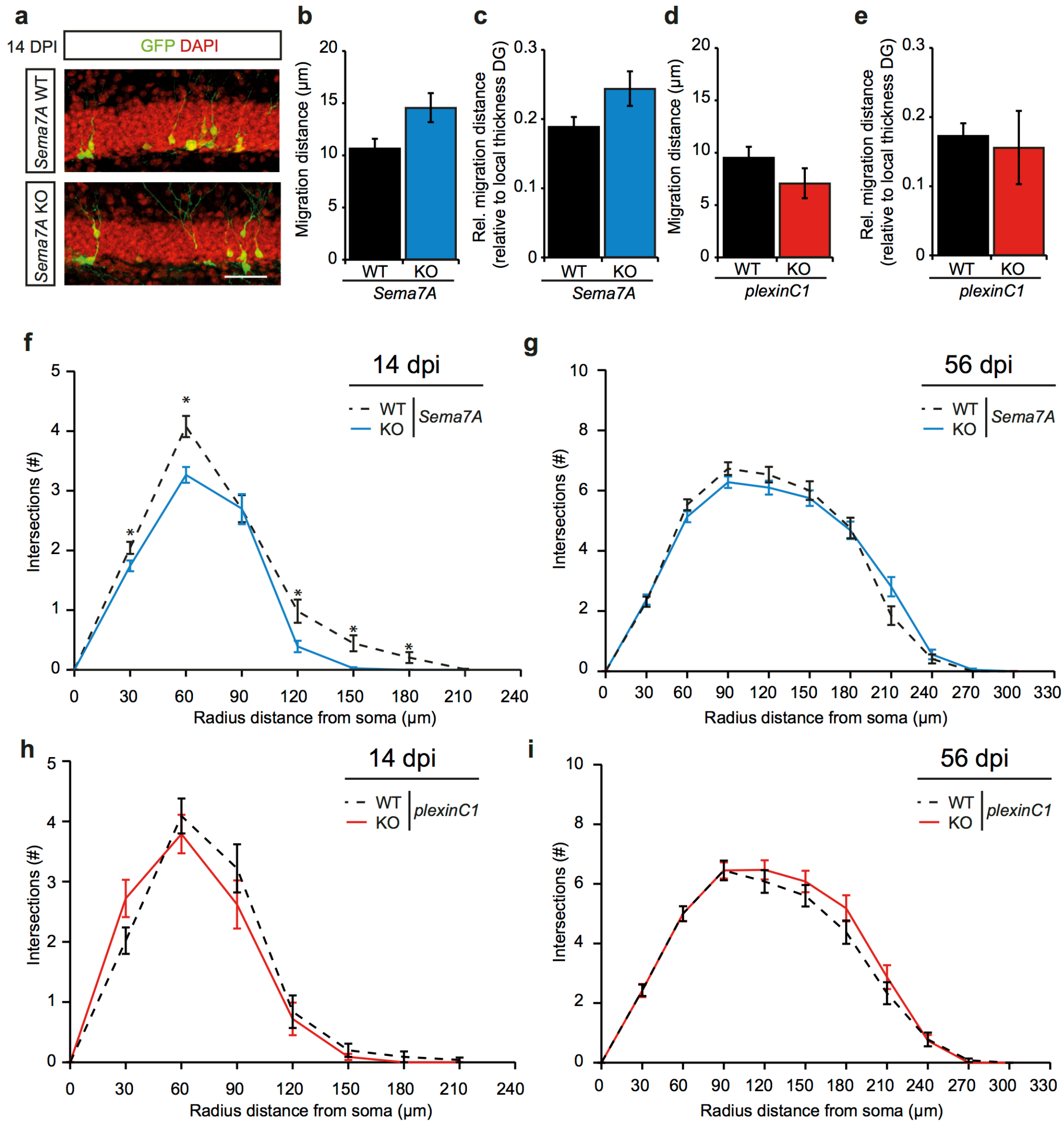

Supplementary Figure 8. Migration of adult-born granule cells in the DG of *plexinC1*$^{-/-}$ and *Sema7A*$^{-/-}$ mice is intact but Sema7A is required for early stages of dendrite development during adult neurogenesis.

(**a**) Representative examples of adult-born cells infected with MMLV CAG-GFP and visualized at 14 days post-infection (dpi) in *Sema7A*$^{-/-}$ (KO) and *Sema7A*$^{+/+}$ (WT) mice.

(**b, d**) Quantification of the migration of adult-born granule cells as shown in **a** (distance between the edge of subgranular zone (SGZ) and center of GFP-positive cell) in KO (n = 4) and WT (n = 4) mice (Student's t-test $P_{(Sema7A)}$ = 0.1645, $P_{(plexinC1)}$ = 0.2801).

(**c**, **e**) Quantification of the migration of adult-born granule cells relative to the local width of the SGZ (Student's t-test: $P_{(plexinC1)}$ = 0.5265).

(**f-i**) Sholl-analysis of the dendritic trees of adult-born granule cells at 14 or 56 dpi with MMLV CAG-GFP in *Sema7A*$^{-/-}$ (KO), *plexinC1*$^{-/-}$ (KO) and wild-type littermates (WT) mice. (Sema7A: KO, n = 120 cells/3 mice (**f**), n = 79 cells/3 mice (**g**), WT, n = 117/4 mice cells (**f**), 60 cells/4 mice (**g**). **f**: repeated-measures ANOVA $F_{(7, 1645)}$ = 2.204, **g**: repeated-measures ANOVA $F_{(9, 1224)}$ = 2.202, P = 0.082. plexinC1: KO, n = 47 cells/ 4 mice (**h**), n = 38 cells/5 mice (**i**), WT, n = 45 cells/4 mice (**h**), 40 cells/5 mice (**i**). **h**: repeated-measures ANOVA $F_{(7, 630)}$ = 1.362 P = 0.219, **i**: repeated-measures ANOVA $F_{(9, 684)}$ = 0.815 P = 0.510). Whiskers represent SEM. *P < 0.05. Scale bar: 50 µm.

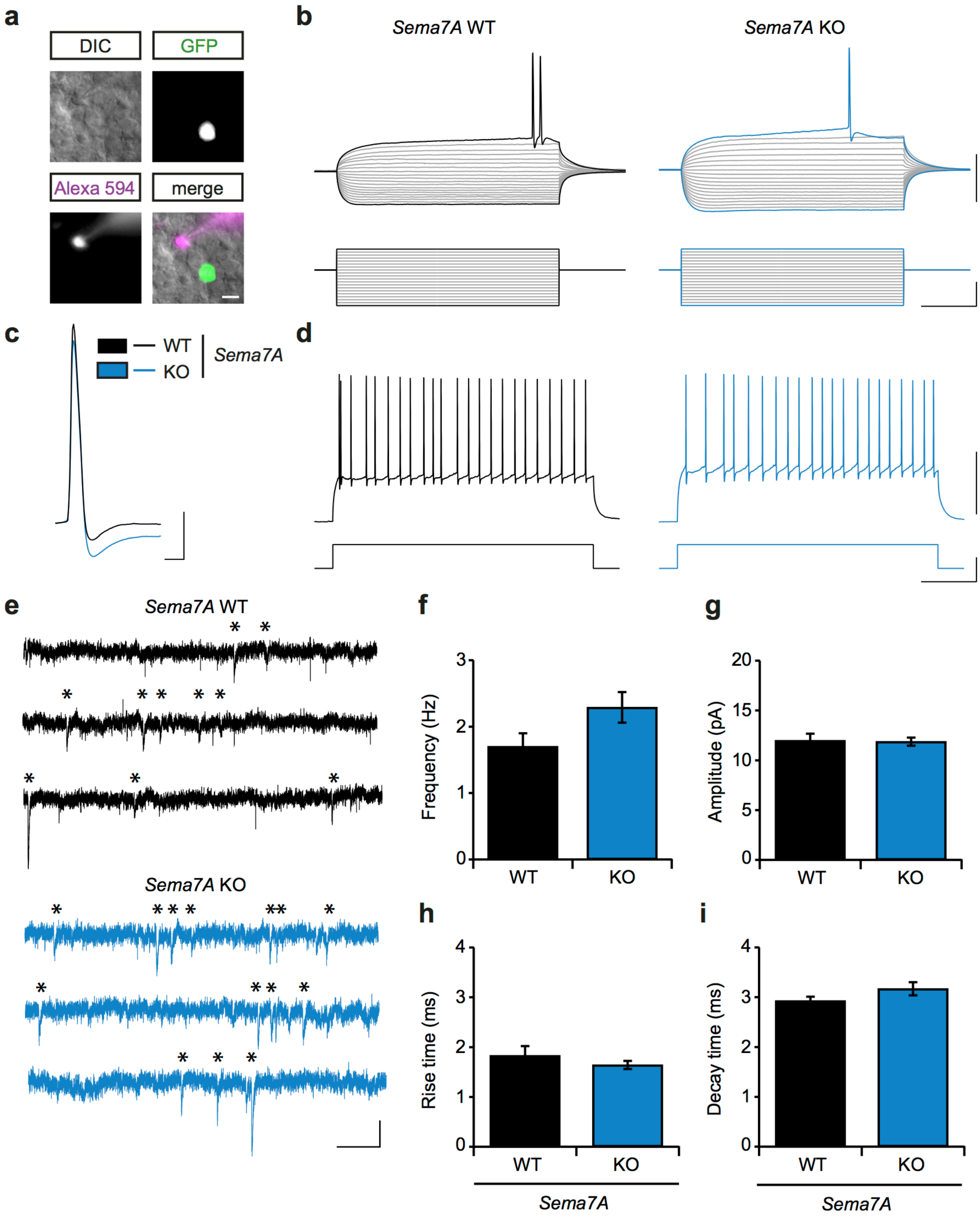

Supplementary Figure 9. Electrophysiological characterization of mature granule cells in the DG of Sema7A knockout mice.

(**a**) Representative image of a mature granule cell (GC) used for recording of electrophysiological properties and miniature excitatory post-synaptic currents (mEPSCs). Healthy cells in the outer layer of the granule cell layer were identified using differential interference contrast (DIC). Recording of mature GCs (GFP-negative cells) was confirmed by the presence of recording pipet-dye Alexa Fluor 594. (**b**) Representative traces of membrane potential (upper traces) in response to current injections (lower traces) in *Sema7A*$^{+/+}$ (WT, black) or *Sema7A*$^{-/-}$ (KO, blue) mature GCs.

(**c**) Representative traces of action potentials in WT and KO nGCs.

(**d**) Representative traces for action potential firing rates (upper trace) in response to a 200 pA current injection (lower trace) in WT and KO mature GCs.

(**e**) Representative traces of mEPSC recordings in WT and KO mature GCs. Asterisks indicate identified mEPSC events.

(**f-i**) Quantification of mEPSC instantaneous frequency (**f**), amplitude (**g**), rise- (h) and decay- (**i**) time ($n_{(WT)}$ = 6 cells/3 mice, $n_{(KO)}$ = 9 cells/4 mice, Student's *t*-test: $P_{(instantaneous\ frequency)} = 0.091$, $P_{(amplitude)} = 0.886$, $P_{(rise\ time)} = 0.307$, $P_{(decay\ time)} = 0.201$). Scale bars: **a** 10 µm, **b**, **d** horizontal: 220 ms, vertical: 50 mV (upper traces) or 200 pA (bottom traces), **c** horizontal: 2 ms, vertical: 20 mV, **e** horizontal: 200 ms, vertical: 10 pA. All data are presented as means ± SEM.

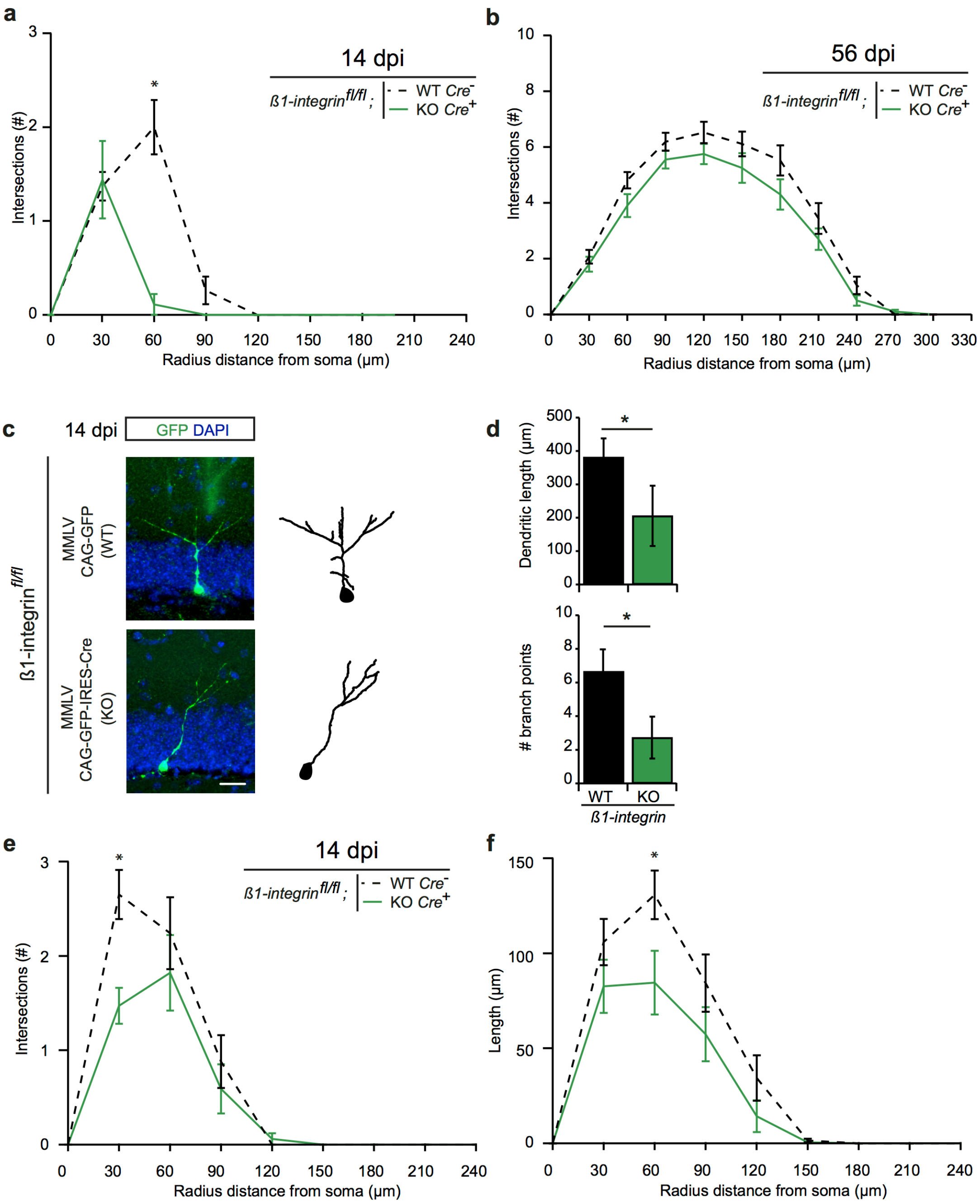

Supplementary Figure 10. Reduced dendritic complexity in *ß1-integrin*$^{-/-}$ adult-born granule cells.

(**a**, **b**) Sholl-analysis of the dendritic trees of adult-born granule cells (nGCs) at 14 or 56 days post-injection (dpi) with MMLV CAG-GFP-Cre and MMLV CAG-RFP in *ß1-integrin*$^{fl/fl}$ mice. (14 dpi: KO, n = 7 cells/4 mice, WT, n = 20 cells/4 mice; 56 dpi: KO, n = 20 cells/4 mice, WT, n = 27 cells/4 mice. **a**: repeated-measures ANOVA $F_{(7,175)} = 2.822$ $P < 0.0001$, **b**: repeated-measures ANOVA $F_{(9, 405)} = 0.783$ $P = 0.513$). Asterisk in **a** represents significant difference (Student's *t*-test: *$P < 0.05$). WT, *ß1-integrin*$^{fl/fl}$ + MMLV CAG-RFP; KO, *ß1-integrin*$^{fl/fl}$ + MMLV CAG-GFP-Cre and MMLV CAG-RFP. Cell-autonomous knockout of ß1-integrin in adult-born granule cells reduces dendritic complexity at 14 dpi.

(**c**) Representative z-stack projection from confocal images of MMLV CAG-GFP-IRES-Cre or CAG-GFP infected nGCs in *ß1-integrin*$^{fl/fl}$ mice. Right panel depicts corresponding tracings.

(**d**) Quantification of dendritic length (top panel) and number of branch points (bottom panel) of KO nGCs (*ß1-integrin*$^{fl/fl}$ + MMLV CAG-GFP-IRES-Cre, green bars, n = 4 mice) compared to WT nGCs (*ß1-integrin*$^{fl/fl}$ + MMLV CAG-GFP, black bars, n = 4 mice) shows decreased dendritic length and branch points (Student's *t*-test: *$P < 0.05$ and **$P < 0.01$) following cell-autonomous loss of *ß1-integrin*. Scale bar: 25 µm.

(**e-f**) Sholl-analysis of the dendritic tree of nGCs at 14 dpi with MMLV CAG-GFP-IRES-Cre (KO), green lines, n = 17 cells/4 mice, and MMLV CAG-GFP (WT), dashed lines, n = 26 cells/4 mice. (**e**: number of intersections per radius distance from soma (30 µm radius interval), repeated-measures ANOVA: $F_{(7, 287)} = 7.09$, $P < 0.0001$. **f**: dendritic length per radius distance from soma, repeated-measures

ANOVA: $F_{(7, 287)} = 6.898$, $P < 0.0001$). Asterisks in **e** and **f** represent significant difference (Student's *t*-test: $^{*}P < 0.05$ and $^{**}P < 0.001$). All data are presented as means ± SEM.

Supplementary Table 1. Electrophysiological properties of adult-born 56 dpi GCs in *Sema7A*$^{+/+}$ and *Sema7A*$^{-/-}$ mice.

Electrophysiological property	*Sema7A* WT	*Sema7A* KO	Statistical significance (*P*)
Resting potential (mV)	-69.59 (1.11)	-68.43 (0.97)	0.4664
Input resistance (MOhm)	245.12 (11.49)	197.47 (9.63)	0.0069*
Time constant (ms)	13.63 (0.92)	11.10 (1.06)	0.1304
Membrane capacitance (pF)†	56.61 (5.00)	55.18 (2.78)	0.7886
Hyperpolarization (mV)§	-134.97 (2.35)	-123.03 (1.85)	0.0011*
Sag (mV)§§	1.78 (0.41)	1.42 (0.16)	0.3354
SagArea (mV•ms)	60.34 (20.98)	40.93 (5.77)	0.2686
Action potential property			
Threshold (mV)	-33.72 (0.93)	-33.93 (1.21)	0.9069
Threshold (pA)	120.56 (6.61)	151.45 (7.95)	0.0167*
Amplitude (mV)	68.00 (3.62)	65.10 (4.03)	0.6191
Width (ms)	2.50 (0.24)	2.72 (0.13)	0.4078
Rise time (ms)	0.86 (0.08)	0.86 (0.05)	0.9725
Fall time (ms)	1.54 (0.16)	1.76 (0.10)	0.2473
Afterhyperpolarization (mV)	9.43 (1.10)	6.20 (0.89)	0.0359
Firing rate (Hz)‡	25.07 (2.95)	27.07 (4.27)	0.7311
Firing adaptation index‡‡	0.32 (0.09)	0.17 (0.12)	0.3801

Data are presented as means with SEM between parentheses. Statistical significance (*P*) was calculated with Student's *t*-test. $n_{Sema7A\ \mathrm{WT}}$ = 7 cells/3 mice, $n_{Sema7A\ \mathrm{KO}}$ = 13 cells/3 mice.

* *P* < Benjamini-Hochberg (BH) critical value after correction for multiple testing, BH-correction, FDR = 0.1.

† Calculated from the time constant and input resistance, as $C_m = \tau_m/R_{in}$

§ Steady-state hyperpolarization, measured in response to a -300 pA current injection step

§§ Initial hyperpolarization overshoot relative to steady-state hyperpolarization§

‡ Calculated from the averaged inter-spike-interval in response to a 200 pA current injection step

‡‡ Calculated from the averaged last three inter-spike-intervals (F_{last}) and first two inter-spike-intervals ($F_{initial}$), as $1-(F_{last}/F_{initial})$

Supplementary table 2. Electrophysiological properties of mature GCs in *Sema7A*$^{+/+}$ and *Sema7A*$^{-/-}$ mice.

Electrophysiological property	*Sema7A* WT	*Sema7A* KO	Statistical significance (*P*)
Resting potential (mV)	-71.14 (1.22)	-72.76 (1.15)	0.3471
Input resistance (MOhm)	185.73 (18.43)	205.79 (12.36)	0.3596
Time constant (ms)	12.56 (1.34)	13.17 (0.89)	0.6956
Membrane capacitance (pF) †	68.04 (3.76)	65.48 (4.45)	0.6733
Hyperpolarization (mV)§	-122.71 (4.26)	-128.42 (2.99)	0.2697
Sag (mV)§§	1.64 (0.25)	1.85 (0.20)	0.5191
SagArea (mV•ms)	59.25 (10.01)	62.12 (8.04)	0.8226
Action potential property			
Threshold (mV)	-36.11 (2.25)	-33.81 (0.95)	0.3204
Threshold (pA)	151.14 (11.04)	143.26 (8.97)	0.5803
Amplitude (mV)	70.52 (5.05)	76.08 (2.19)	0.2981
Width (ms)	3.28 (0.89)	2.25 (0.15)	0.2293
Rise time (ms)	0.80 (0.06)	0.75 (0.04)	0.5559
Fall time (ms)	2.38 (0.89)	1.39 (0.12)	0.2480
Afterhyperpolarization (mV)	8.25 (1.51)	10.19 (1.30)	0.3376
Firing rate (Hz)‡	23.64 (4.83)	26.40 (3.46)	0.6398
Firing adaptation index‡‡	0.25 (0.12)	0.07 (0.12)	0.3159

Data are presented as means with SEM between parentheses. Statistical significance (*P*) was calculated with Student's *t*-test. $n_{Sema7A\ \mathrm{WT}}$ = 13 cells/4 mice, $n_{Sema7A\ \mathrm{KO}}$ = 16 cells/5 mice.

† Calculated from time constant and input resistance, as $C_m = \tau_m/R_{in}$

§ Steady-state hyperpolarization, measured in response to a -300 pA current injection step

§§ Initial hyperpolarization overshoot relative to steady-state hyperpolarization§

‡ Calculated from the averaged inter-spike-interval in response to a 200 pA current injection step

‡‡ Calculated from the averaged last three inter-spike-intervals (F_{last}) and first two inter-spike-intervals ($F_{initial}$), as $1-(F_{last}/F_{initial})$
